# Enhancing mesothelin CAR T cell therapy for pancreatic cancer with an oncolytic herpes virus boosting CAR target antigen expression

**DOI:** 10.1007/s00262-025-04039-7

**Published:** 2025-05-14

**Authors:** Mona Alhussein Aboalela, Mohamed Abdelmoneim, Shigeru Matsumura, Ibrahim Ragab Eissa, Itzel Bustos-Villalobos, Patricia Angela Sibal, Yu Orikono, Yuhei Takido, Yoshinori Naoe, Hideki Kasuya

**Affiliations:** 1 Cancer Immune Therapy Research Center, Graduate School of Medicine, Nagoya University, Nagoya, 466-8550 Japan; 2 Department of Gastroenterological Surgery, Graduate School of Medicine, Nagoya University, Nagoya, 466-8550 Japan; 3 Department of Medical Microbiology and Immunology, Faculty of Medicine, Zagazig University, Zagazig, 44519 Egypt; 4 Department of Microbiology, Faculty of Veterinary Medicine, Zagazig University, Zagazig, 44519 Egypt; 5 Surgical Oncology Division, Department of Surgery, Massachusetts General Hospital, Harvard Medical School, Boston, MA 02114 USA; 6 Department of Neurosurgery, Graduate School of Medicine, Nagoya University, Nagoya, 466-8550 Japan

**Keywords:** Mesothelin, CAR T cell therapy, Oncolytic virus, Herpes virus, Pancreatic cancer, Syngeneic murine model

## Abstract

**Abstract:**

Mesothelin (MSLN) is a prominent target antigen for CAR T cell therapy due to its extensive expression in various solid tumors, including pancreatic cancer. However, the therapeutic efficacy of MSLN-targeted CAR T cell therapy has been limited in clinical trials for pancreatic cancer, often resulting in temporary stable disease as the best response. The heterogeneous expression of MSLN and its loss over time, along with the immunosuppressive tumor microenvironment (TME), are key factors restricting effectiveness. Oncolytic viruses are emerging cancer therapies that replicate in tumor cells and remodel the TME into an immunogenic state. Here, we engineered an oncolytic herpes simplex virus type 1 expressing human MSLN (HSV-MSLN) and evaluated its combination with MSLN-CAR T cells in a murine pancreatic ductal adenocarcinoma model. In vitro, HSV-MSLN effectively induced MSLN expression on murine pancreatic cancer cells, with subsequent cell lysis. In co-culture, HSV-MSLN-infected cancer cells activated MSLN-CAR T cells, which effectively eliminated the infected cells. In vivo, HSV-MSLN delivered MSLN on the tumor cell surface and reprogrammed the TME toward an immunogenic state. The combination therapy significantly enhanced antitumor efficacy, inducing activated, proliferative CD8^+^ CAR T cells and reducing PD-1^+^TIM-3^+^ exhausted endogenous CD8^+^ T cells and regulatory T cells in tumors. Furthermore, the combination therapy increased migratory XCR1^+^CD103^+^ dendritic cells (DCs) in tumors and tumor-draining lymph nodes (TDLNs) while expanding CD44^+^CD8^+^ T cells with central and effector memory phenotypes. Taken together, these results demonstrate that HSV-MSLN reprograms immune cells in the TME and TDLNs and synergizes with MSLN-CAR T cells to enhance antitumor responses, leading to a more robust therapeutic effect.

**Graphical abstract:**

MSLN-CAR T cell therapy showed limited efficacy against pancreatic cancer due to heterogeneous antigen expression and an immunosuppressive TME. Here, we engineer the oncolytic virus HSV-MSLN to deliver MSLN and reshape the TME, combining it with MSLN-CAR T cells. Combination therapy reduces tumor burden, enhances CAR T cell activation and proliferation, and decreases exhaustion. Additionally, it increases CD4^+^ and CD8^+^ T cell infiltration, enhances CD8^+^ T cell activation, reduces exhaustion, and decreases Tregs in the tumor. It also elevates migratory DCs in tumors and TDLNs and expands central and effector memory CD8⁺ T cells, promoting a dynamic immune cycle that amplifies antitumor immunity.
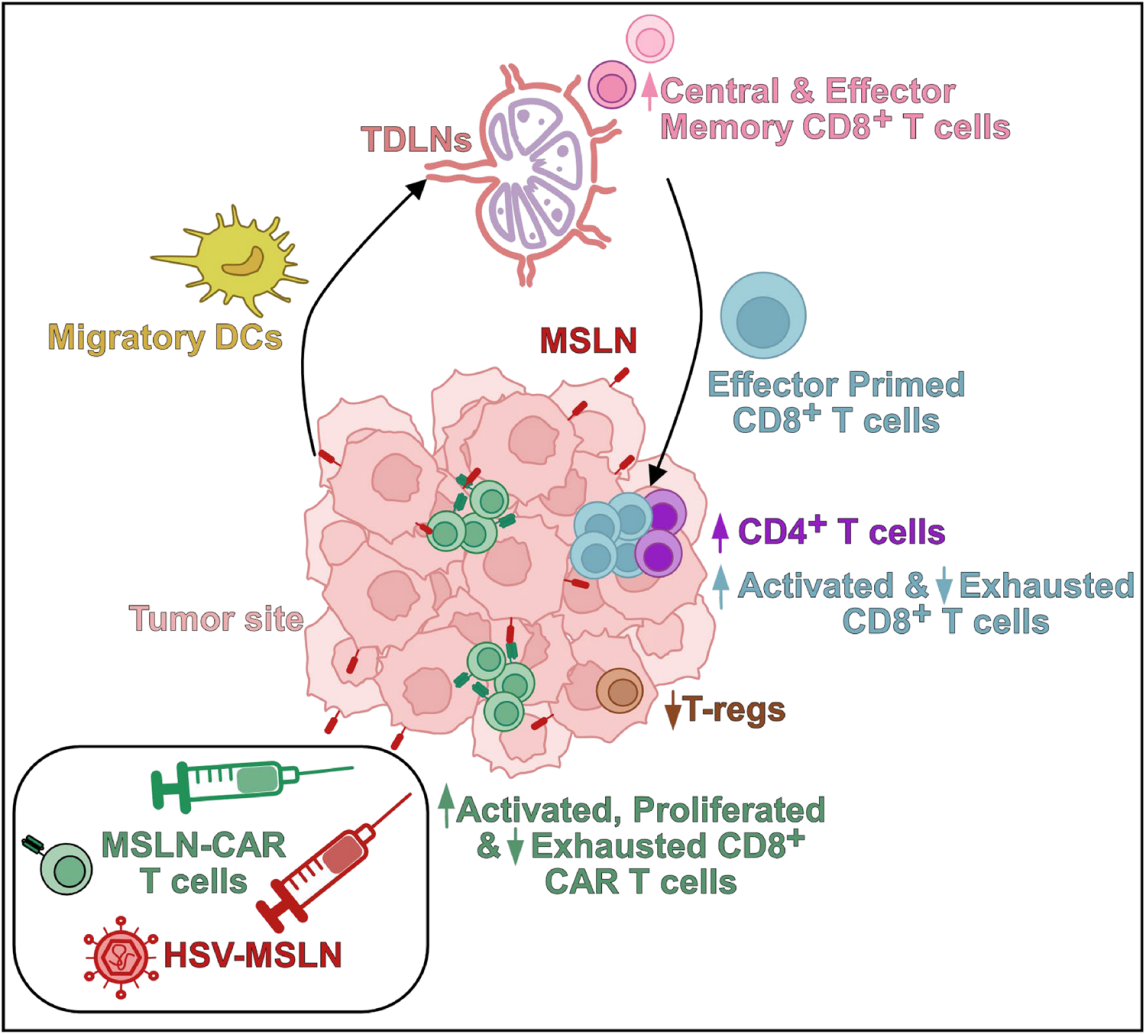

**Supplementary Information:**

The online version contains supplementary material available at 10.1007/s00262-025-04039-7.

## Introduction

Pancreatic cancer is a highly aggressive and lethal solid tumor and ranks as the 6th leading cause of cancer deaths worldwide. The most common subtype is pancreatic ductal adenocarcinoma (PDAC), accounting for 85–90% of cases [1]. Despite surgical advances, only 15–20% of patients are diagnosed early enough for surgery, making chemotherapy the primary treatment for most patients [2]. In recent years, immunotherapy, such as adoptive T cell (ATC) therapies, oncolytic viruses (OVs), immune checkpoint blockades (ICBs), and bispecific T cell engagers (BiTEs), has significantly transformed oncology and holds promise for treating solid tumors, including PDAC [3]. While ICBs have shown good effects in some solid tumors, their effect in PDAC is limited [4]. ICBs work by reversing immune suppression through the activation of pre-existing T cell immunity, but PDAC lacks sufficient pre-existing T cell infiltration (CD8^+^ and CD4^+^ T cells) [5]. Similarly, BiTEs, which are designed to bind T cells and tumor cells together, face the same limitations in PDAC due to insufficient T cells in the tumor microenvironment (TME) [4]. Additionally, innate immune cells, natural killer (NK) cells, and dendritic cells (DCs) are present in low numbers or lack the necessary activation receptors and maturation markers, while immunosuppressive T regulatory cells (Tregs) are highly infiltrated [6–9]. Collectively, these factors make the TME in PDAC highly immunosuppressive, with impaired T cell priming and function.

Chimeric antigen receptor (CAR) T cell therapy, a form of ATC therapies that involves genetically modifying T cells with CARs to target tumor-associated antigens (TAAs), has demonstrated significant attention due to its promising clinical outcomes in hematological malignancies, mainly targeting CD19 and B-cell maturation antigen (BCMA), leading to Food and Drug Administration (FDA) approval for B-cell malignancies [10]. However, translating this success to solid tumors, particularly PDAC, faces difficulties. One obstacle is the homing of CAR T cells to tumor sites in solid tumors; for instance, PDAC has a physical barrier of dense desmoplastic stroma, which makes up 70–90% of the PDAC, which impedes CAR T cells diffusion [11, 12]. Additionally, after homing, the survival and proliferation of the CAR T cells are highly affected by the immunosuppressive TME of the PDAC [12].

Another challenge is the selection of a target antigen for CAR T cell therapy in solid tumors. There are several antigens under study for PDAC, of which mesothelin (MSLN) stands out as the most advanced. MSLN is a TAA prevalent in various solid tumors, approximately 40% of solid tumors, while having minimal presence in normal tissues [13, 14]. It is expressed in about 80% of pancreatic cancer patients, while absent in normal pancreas and chronic pancreatitis [15, 16]. These characteristics highlight MSLN’s potential in current clinical trials investigating MSLN-targeted therapies, including CAR T cells [17]. MSLN-CAR T cell therapy has been investigated in 15 clinical trials for pancreatic cancer. However, utilizing MSLN-CAR T cells in solid tumors, particularly pancreatic cancer, has shown limited efficacy, with stable disease being the best outcome observed [18, 19]. One reason for this limited efficacy is the heterogeneity in MSLN target expression on tumor cell surfaces. Over time, the CAR target—particularly MSLN—can be significantly downregulated or shed from the tumor cell surfaces (temporal heterogeneity), and its expression can also vary between different tumor cells within the same patient (spatial heterogeneity) [20]. Considering the density of antigen expression, even the level of MSLN expression can be detectable but scattered within a tumor, meaning the tumor is technically MSLN-positive, but the antigen density may not be enough to activate MSLN-CAR T cells effectively. Hence, increasing MSLN antigen density could be a key for maximizing the effectiveness of MSLN-targeted CAR T cell therapy.

To overcome the limitations of MSLN-CAR T cell therapy, supplementary treatments that can modify the immunosuppressive TME and address the antigen density challenge can be used. Oncolytic virotherapy is an immunotherapy in which naturally or genetically modified viruses selectively kill tumor cells while sparing healthy cells [21]. OVs selectively replicate within tumor cells, causing them to release TAAs, cellular damage-associated molecular patterns (DAMPs), and pathogen-associated molecular patterns (PAMPs), stimulating the antitumor immune response [22]. Two genetically engineered oncolytic herpes virus type 1 (oHSV1) have been approved: talimogene laherparepvec (T-VEC) for melanoma by the FDA in the USA in 2015 and G47Δ for glioblastoma in Japan in 2022 [23, 24]. Clinical and preclinical studies have shown the potential therapeutic effects of oHSV1 in treating PDAC [25]. These studies indicate that oHSV1 successfully softened the dense PDAC tumor tissue and altered the immunogenetically cold TME to hot [26, 27]. Furthermore, oHSV1 can be genetically engineered to introduce specific proteins onto the surface of tumor cells [28]. Considering the potential to introduce robust antigen density (MSLN) on tumor cells and enhance the antitumor immune response, there is a strong rationale for combining oHSV1-expressing CAR-target antigen with CAR T cell therapy. It has not yet been explored whether MSLN expression via an OV can further improve the antitumor activity of MSLN-targeted CAR T cells. In this study, we generated an HSV-MSLN from a clinical HSV1 strain and combined it with MSLN-CAR T cell therapy against murine PDAC. The HSV-MSLN effectively delivered MSLN to pancreatic cancer cells in vitro and activated MSLN-CAR T cells when co-cultured. In vivo, combining HSV-MSLN with MSLN-CAR T therapy resulted in a significant suppression of tumor growth and increased survival in the murine PDAC tumor model. Mechanistically, HSV-MSLN reprogrammed the TME by increasing the infiltration of unexhausted CD8⁺ T cells, reducing Tregs, and enhancing MSLN-CAR T cells activation and proliferation compared to MSLN-CAR T cell monotherapy. This synergistic effect also promoted DC activation and migration, improving antigen presentation and priming of naïve CD8⁺ T cells in tumor-draining lymph nodes (TDLNs). These findings underscore the promise of combining oncolytic virotherapy with CAR T cells to advance immunotherapy for solid tumors.

## Materials and methods

### Cell lines

Murine PDAC cell lines Pan02 (RRID: CVCL_D627) and KPC (RRID: CVCL_XD12) were kindly provided by Dr. Sho (Nara Medical University) and Dr. Ohuchida (Kyushu University), respectively. African green monkey kidney cells (Vero cells; RRID: CVCL_0059) and PLAT-E cells (RRID: CVCL_B488) were obtained from the American Type Culture Collection (Manassas, VA, USA) and Cell Biolabs Inc. (San Diego, CA, USA), respectively.

All cell lines were cultured in Dulbecco’s Modified Eagle Medium with high glucose (Wako, Osaka, Japan), supplemented with 10% heat-inactivated fetal bovine serum (FBS; F7524, Sigma, Tokyo, Japan), and 100 IU/mL of penicillin/streptomycin (Wako) at 37 °C in a humidified atmosphere containing 5% CO_2_. For PLAT-E cells, the medium was additionally supplemented with 10 µg/mL blasticidin and 1 µg/mL puromycin (both from Wako, Osaka, Japan).

Pan02-MSLN and KPC-RFP cells were generated by transfecting Pan02 or KPC cells with MSLN-coding retrovirus or RFP-coding retrovirus, harvested from supernatants of PLAT-E cells transfected with MSLN-pMX-GFP or pMX-RFP, respectively.

### HSV-MSLN generation

HSV1 was isolated from patients and kindly provided by Dr. Takashi Kawana at Teikyo University, Japan. For the generation of HSV-MSLN, the CMV promoter with the human MSLN-coding region and the γ34.5 region of HSV1 for homologous arms were cloned into the pBluescript plasmid. The HSV1 virus genome and the linearized pBluescript MSLN-γ34.5 target vector were transfected into Vero cells using lipofectamine LTX with plus reagent. Afterward, the plaque purification process was performed until the purity of HSV-MSLN was confirmed by quantitative polymerase chain reaction (q-PCR). Viral titers were assayed in Vero cells and expressed as plaque-forming units per milliliter (PFU/mL) and stored at − 80°C.

### q-PCR for HSV-MSLN purity assessment

To assess the purity of the HSV-MSLN virus, HSV-MSLN DNA was first isolated using the phenol–chloroform extraction method. q-PCR was performed using the THUNDERBIRD Probe q-PCR Mix (TOYOBO) on a StepOne Real-Time PCR System (Thermo Fisher Scientific) for DNA amplification under the following cycling conditions: 50 °C for 2 min, 95 °C for 2 min, followed by 40 cycles of 95 °C for 15 s, and 60 °C for 1 min. Three PrimeTime^™^ q-PCR probe-primer sets (Integrated DNA Technologies) were designed to target: (1) the US4 region (present in all HSV strains) as an internal control; (2) the CMV promoter (in the cassette inserted into HSV-MSLN); and (3) the γ34.5 region (deleted in HSV-MSLN) (Table.S1). The q-PCR analysis was performed using the StepOne real-time PCR System software, including standards ranging from 6 × 10^6^ to 60 copies per reaction and negative controls. These standards were used to generate a standard curve and calculate the copy number of unknown samples.

### MSLN expression on infected cancer cells

Pan02 cells and KPC were infected with HSV-MSLN at (0, 0.125, 0.25, 0.5, and 1) multiplicity of infections (MOIs). After 18 h, cells were harvested and stained with the Biotin anti-human MSLN antibody clone MB (BioLegend) at 4 °C for 30 min. Cells were subjected to flow cytometer, FACSCanto II (BD Biosciences, San Diego, CA). Data were analyzed using the FlowJo software (FlowJo, Ashland, OR).

### Cell proliferation assay

Cell proliferation was determined using the 3-(4,5-dimethylthiazol-2-yl)-2,5-diphenyl tetrazolium bromide (MTT) dye reduction method. Pan02 cells and KPC cells were seeded on 96-well plates and incubated for 24 h at 37 °C with 5% CO_2_. After 24 h, cells were infected with HSV-MSLN at MOIs of 0.25, 0.5, or 1 for 24, 48, or 72 h. Viable cells were quantified using colorimetric MTT assays.

### Virus replication assay

Pan02 and KPC were seeded and incubated at 37 °C with 5% CO_2_. After 24 h, cells were infected with 1 MOI of HSV-MSLN. Cells and supernatant are harvested at 24, 48, and 72 h post-infection and lysed with three freeze–thaw cycles for titration. Viral titers were assayed in Vero cells and expressed as PFU/ml.

### DCs maturation assay

For bone marrow-derived dendritic cell (BMDC) generation, bone marrow cells were isolated from the femurs and tibiae of female C57BL/6 mice. The cells were cultured in complete RPMI medium supplemented with 10% FBS, 100 IU/mL penicillin/streptomycin (Wako), 50 mM 2-mercaptoethanol (Gibco), 1% HEPES (1 M) (Thermo Fisher), and recombinant murine GM-CSF (rmGM-CSF; 20 ng/mL; R&D Systems, Minneapolis, MN). The same volume of culture medium was added on day 3. On day 6, the cells were checked with DC markers (CD11b^+^/CD11c^+^) to ensure differentiation into BMDCs. Afterward, BMDCs were split into 24-well non-treated plates (10^6^ cells/well) and cultured with RPMI-based conditioned medium of Pan02 cells which were treated respectively for 24 h. After 1 day incubation with conditioned medium, DCs were harvested and checked by CD11c, CD11b, CD86, CD40, and MHCII with the flow cytometer.

### T cells activation and cytotoxicity

For T cells activation, Pan02 and KPC were infected with HSV-MSLN at 0.25, 0.5, and 1 MOIs. After 24 h, MSLN-CAR T cells were added at an effector target (ET) ratio of 1:1. After 6 h, cells were harvested and stained for intracellular IFN-γ (2 μM monensin was added 3 h before cell harvesting) and surface CD45, CD69, and CD25 antibodies (BioLegend) at 4°C for 30 min. For intracellular staining, cells were fixed using 4% Paraformaldehyde Phosphate Buffer Solution (Wako, Osaka, Japan) and permeabilized using 0.5% Polyoxyethylene (10) Octylphenyl Ether (Wako, Osaka, Japan), then stained with IFN-γ antibody for 30 min at 4 °C in the dark. Cells were subjected to flow cytometer on a FACSCanto II (BD Biosciences, San Diego, CA). Data were analyzed using the FlowJo software (FlowJo, Ashland, OR).

For cell cytotoxicity, Pan02 was infected with HSV-MSLN at 0.5 MOI. After 24 h, mock-T cells or MSLN-CAR T cells were added at an ET ratio of 1:1. After 24 h, cell death was visualized by phase-contrast microscopy.

To monitor the kinetics of the cytotoxicity of MSLN-CAR T cells, the fluorescent time-lapse imaging analysis was performed to monitor RFP-expressing KPC cells. KPC-RFP cells were infected or not infected with HSV-MSLN at a MOI of 0.5. After 24 h, MSLN-CAR T cells or mock-T cells were added at an ET ratio of 1:1, and cell death was monitored by measuring the reduction in RFP signals. Time-lapse imaging of the live cells during incubation was performed using an All-in-One Fluorescence Microscope (BZ-X800, KEYENCE, Japan). Images were captured every hour for 36 h at least 3 sites per well using a 20X objective lens, with the cells maintained at 37°C and 5% CO_2_. The red area was quantified using the BZ-X800 analyzer software (Keyence). Initially, the haze reduction under the same conditions was applied to the acquired time-lapse images. To determine the red area, a certain threshold was set. The total red area in an image of each timepoint was then calculated by the software.

### Murine T cells isolation and CAR T cells generation

Murine T cells were isolated from the spleens of C57BL/6J/CD45.2 mice, C57BL/6 J/CD45.1 mice, and C57BL/6-Tg (TcraTcrb)1100Mjb/J (OT-1) mice, which were purchased from Japan SLC (Hamamatsu, Japan), Sankyo Laboratory (Tokyo, Japan) and The Jackson Laboratory (stock number 003831), respectively. Mice were kept under constant temperature and humidity conditions and fed with a standard diet and water. All mice were maintained under specific pathogen-free conditions. All experiments were reviewed and approved by the Animal Care University Committee following the Guidelines for Animal Experimentation at Nagoya University (M220181, M230153, and M240255) (Nagoya, Japan). All methods were carried out in accordance with relevant guidelines and regulations. All methods are reported in accordance with ARRIVE guidelines. C57BL/6 J/CD45.1 mice spleen was used as T cells donors only in the tumor-infiltrating lymphocytes (TILs) analysis experiment.

T cells were isolated by MACS magnetic cell sorting following the manufacturer’s instructions and activated for 24 h using anti-mouse CD3ε (Clone 145-2C11) and anti-mouse CD28 (Clone 37.51) (BioLegend). T cells were maintained in RPMI media supplemented with 10% FBS, 100 IU/mL of penicillin/streptomycin (Wako), 50 mM 2-mercaptoethanol (Gibco), 1% MEM non-essential amino acids (Wako), 1% sodium pyruvate (100 mM) (Thermo Fisher), 1% HEPES (1 M) (Thermo Fisher), and 10 ng/mL each of recombinant mouse IL-2 (BioLegend) and IL-7 (Sino Biological).

A murinized second-generation MSLN-CAR was constructed using the human anti-MSLN SS (AF035617.1) scFv, which specifically recognizes human MSLN but not murine MSLN. The scFv was linked to a hinge and transmembrane domain derived from the extracellular and transmembrane regions of human CD28, followed by intracellular signaling domains derived from the cytoplasmic regions of human CD28 and CD3ζ. The complete CAR construct was cloned into the pMXs-IRES-GFP retroviral vector. The MSLN-CAR plasmid was transduced into PLAT-E cells using TransIT-293 transfection reagent (Mirus, MIR2704) according to the manufacturer’s protocol. The supernatant containing retrovirus was collected 48- and 72-h post-transfection and centrifuged for 2 h at 2000 × g in non-adherent 24-well plates, which were pre-coated with 25 μg of RetroNectin (Takara Bio) in 1 mL PBS per well for an overnight at 4 °C. After the viral supernatant was removed, the activated 2 × 10^6^ T cells were added with 2 mL of T cell media and centrifuged for 15 min at 500 × g. Transduced T cells were maintained in T cell media for 48–72 h and then used for experiments.

#### Co-culture for assessment of DC maturation and T cell priming

KPC-OVA cells were infected with HSV-MSLN at an MOI of 0.5. Four hours after infection, the virus was inactivated by UV exposure, and the cells were then incubated for a total of 18 h. After incubation, the infected cells were co-cultured with or without MSLN-CAR T cells at E:T ratio of 1:1 for 20 h. The resulting culture supernatants were collected and used to condition immature BMDCs for 6 h. Naïve OT-1 CD8^+^ T cells were then added at a DC: T cell ratio of 1:4 and incubated for 36 h. Flow cytometric analysis of DC maturation (CD86, CD40) and OT-1 T cell priming and activation (CD25, CD44, CD69).

#### Tumor challenge and treatments in mice

Six- to seven-week-old female C57BL/6J/CD45.2 mice were used for the experiment. Pan02 or KPC tumors were cut into 2 mm^3^ cubes. One tumor cube was inoculated into the right flank of each mouse. When the average tumor size reached 150 mm^3^, mice were randomly divided into groups with equal average tumor sizes. According to the experimental design, HSV-MSLN viruses were diluted in 50 μL saline and injected intratumorally (IT) at doses of 1 × 10^6^, 5 × 10^6^, or 1 × 10^7^ PFU. For combination studies, CAR T cells (3 × 10^6^ cells/mouse) were prepared in 50 μL PBS and injected IT two days after the 2nd dose of HSV-MSLN treatment. As for the control group, mice were injected with 50 μL saline solution. Clinical signs, body weight changes, and tumor growth were monitored. Tumor volume was measured twice weekly until study termination. Tumor volume (V) was estimated using the equation V = L × W^2^/2, where L and W are tumor length and width, respectively.

#### Preparation of single-cell suspensions of TILs and flow cytometry

TILs were collected using a gentle MACS Dissociator (Miltenyi Biotec), filtered through a cell strainer (70 μm), and washed three times with FACS buffer (PBS containing 5% FBS and 0.1% sodium azide). The cells were treated with an anti-CD16/CD32 antibody to block Fc receptors. Subsequently, the cells were stained with the following antibodies (BioLegend): Brilliant Violet 510-conjugated anti-CD45, FITC-conjugated anti-CD45.1, FITC-conjugated or Brilliant Violet 421-conjugated anti-CD3, PerCP-Cy5.5-conjugated anti-TCRβ, APC-Cy7-conjugated anti-CD8a, Pacific Blue-conjugated anti-CD4, PE-conjugated anti-NKp46, Brilliant Violet 421-conjugated anti-CD11b, APC-Cy7-conjugated anti-CD11c, APC-conjugated anti-CD103, PerCP-Cy5.5-conjugated or FITC-conjugated anti-I-A/I-E, FITC-conjugated or PerCP-Cy5.5-conjugated anti-Ly6C, PE-conjugated anti-F4/80, PE-conjugated anti-CD69, PE-conjugated or biotinylated anti-CD25 followed by PerCP-streptavidin, PE-conjugated anti-CD62L, APC-conjugated or PerCP-Cy5.5-conjugated anti-CD44, APC-conjugated anti-CD127, PE-conjugated anti-PD-1, APC-conjugated anti-TIM-3, FITC-conjugated anti-CD86, PE-conjugated anti-XCR1, PE-conjugated anti-Ki67, and APC-conjugated anti-Foxp3. For staining Ki67 and Foxp3, the Bioscience™ Foxp3/Transcription Factor Staining Buffer Set (Thermo Fisher) was used for fixation and permeabilization according to the manufacturer’s protocol. The cells were stained for 30 min at 4°C. After extensive washing with FACS buffer, cells were subjected to flow cytometer on a FACSCanto II (BD Biosciences, San Diego, CA). Data were analyzed using FlowJo software (FlowJo, Ashland, OR).

#### Statistical analysis

Statistical comparisons were performed using GraphPad Prism, version 10.2.3 (GraphPad Software). Statistical significance between the two groups was analyzed using Student’s t-test. One-Way ANOVA with Dunnett’s post-test was used to analyze flow cytometer data between more than 2 groups. Two-way ANOVA with Tukey’s multiple comparisons post-test was used for experiments involving the analysis of multiple time points. *P*-values < 0.05 were considered to be statistically significant.

## Results

### HSV-MSLN effectively delivers human MSLN to the infected pancreatic cancer cells and exhibits cytotoxicity in vitro

**Fig. 1:**
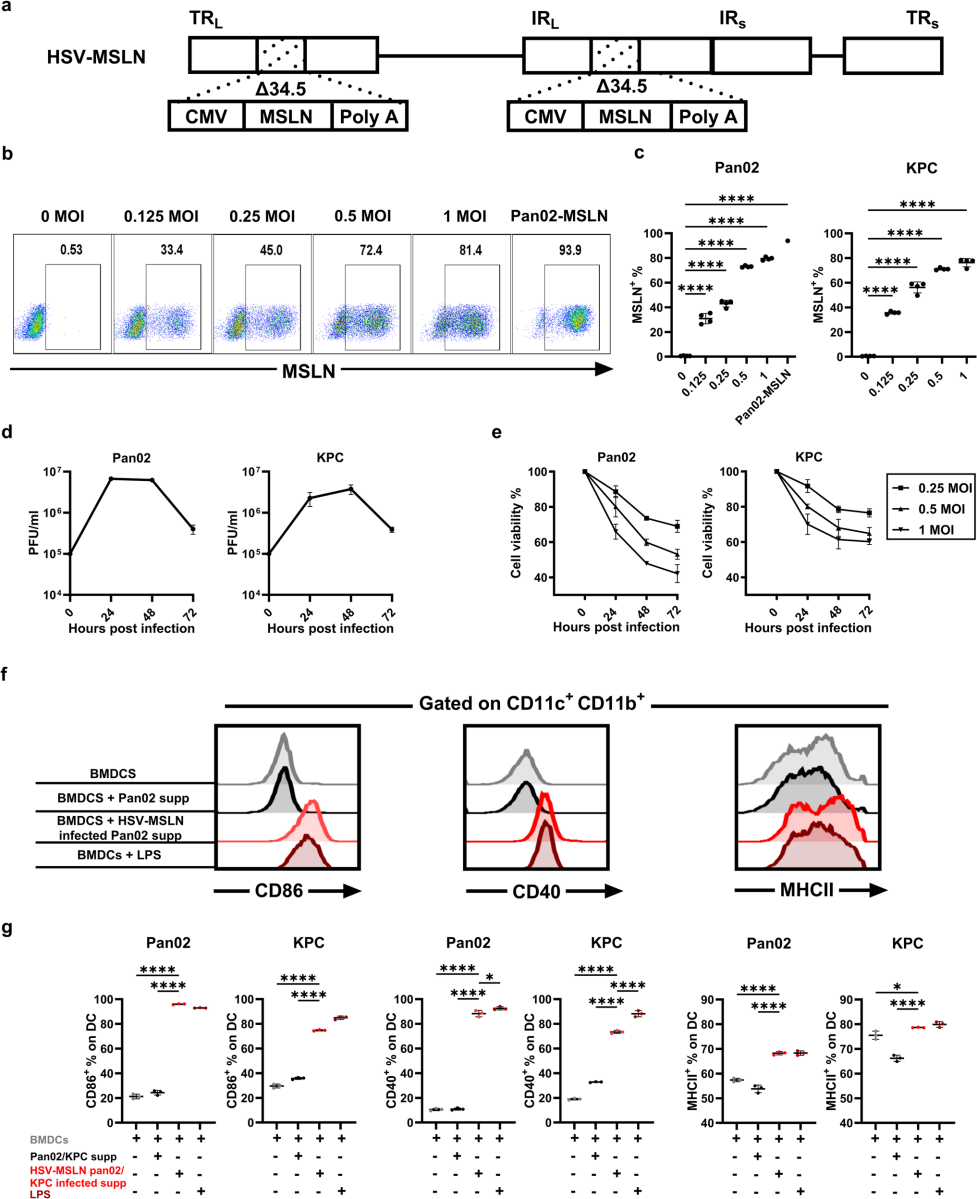
HSV-MSLN effectively induces human MSLN expression on the infected pancreatic cancer cells and exhibits cell cytotoxicity in vitro. **a** Design of HSV-MSLN generation from parent HSV1 by deletion of the two viral γ34.5 neurovirulence genes and insertion of two copies of the human MSLN gene under the control of a CMV promoter. **b** Dot plots from flow cytometer analysis of MSLN-positive Pan02 cells after 18 h of HSV-MSLN infection at indicated MOIs and Pan02-MSLN. Percentages indicate MSLN-positive cells in the boxed gating. **c** Summary of the percentage of MSLN-expressing Pan02 (left) and KPC (right) cell lines after HSV-MSLN infection at the indicated MOI for 18 h. Data are presented as mean ± SD (*n* = 4). One-Way ANOVA followed by Dunnett’s multiple comparison tests were performed. ****p < 0.0001. **d** Viral titer was determined at the indicated time points for Pan02 (left) and KPC cells (right). Data are presented as mean ± SD (*n* = 3). **e** Viability of Pan02 cells (left) and KPC cells (right) after infection with HSV-MSLN at the indicated MOIs, determined by MTT assay at the indicated time points. Data are presented as mean ± SD (*n* = 3). After pancreatic cancer cells were infected with or without HSV-MSLN (10 MOI) for 24 h, the supernatant was collected and added to BMDCs. After 24 h, BMDCs were analyzed for maturation markers (CD86, CD40, and MHCII). **f** Representative histogram from flow cytometer analysis showing the abundance of CD86 (left), CD40 (middle) and MHCII (right) on CD11b^+^ CD11C^+^ BMDCs. **g** Summary of the percentages of CD86^+^ (right), CD40^+^ (middle) and MHCII.^+^ (left). Data are presented as mean ± SD (*n* = 3). One‐Way ANOVA followed by Dunnett’s multiple comparison tests were performed. *p < 0.05, ****p < 0.0001

In this study, HSV-MSLN was constructed using the HSV1 strain 17 backbone. Given its potential to cause illness as a natural human pathogen, genetic modification, and significant attenuation are essential to ensure safety. Therefore, we replace the two viral γ34.5 neurovirulence genes with the human MSLN gene under the control of a CMV promoter to allow for the delivery of MSLN to the pancreatic cancer cell surfaces (Fig. 1a) [29]. The purity of HSV-MSLN was confirmed by q-PCR (Fig. S1). To evaluate HSV-MSLN’s ability to infect and deliver MSLN to pancreatic cancer cell lines, Pan02 and KPC were infected with HSV-MSLN at the indicated MOI for 18 h. The expression of MSLN on the cancer cell surfaces was assessed by flow cytometer. As a positive control, we compared HSV-MSLN-infected cells with Pan02 cells expressing MSLN stably (Pan02-MSLN) (Fig. 1b). MSLN expression increased in a MOI-dependent manner, with nearly 80% of cancer cells showing MSLN positivity after 18 h at a MOI of 1 in both pancreatic cancer cell lines (Fig. 1c). We then assessed the replication functionality of HSV-MSLN in pancreatic cancer cell lines. Pan02 and KPC cells were infected with HSV-MSLN at a MOI of 1, and replication efficiency was evaluated over the indicated time using viral plaque assays. HSV-MSLN demonstrated replication capabilities in both pancreatic cancer cell lines (Fig. 1d). Next, we evaluated the cytotoxic effect of HSV-MSLN using MTT assays. Pan02 and KPC cells were infected at the indicated MOIs, and cell viability was evaluated after 24, 48, or 72 h. HSV-MSLN exhibited cytotoxicity in both a MOI- and time-dependent manner (Fig. 1e). It is well known that the OV-induced cytotoxicity and inflammation release mediators that can enhance DC maturation and immune system in vivo [30–33]. To know if the HSV-MSLN-mediated pancreatic cancer cell death is similarly immunogenic, we checked the maturation status of BMDCs after incubation with conditioned medium from virus infected tumor cells. After Pan02 and KPC cells were infected with or without HSV-MSLN for 24 h, the supernatant was added to BMDCs. Compared to control BMDCs (untreated), BMDCs cultured in the supernatant from HSV-MSLN-treated cells showed a significant upregulation of maturation markers, including CD86, CD40, and MHCII (Fig. 1f, g), which were comparable to those cultured with LPS-conditioned media. In contrast, BMDCs exposed to supernatant from untreated pancreatic cancer cells displayed no response, which is similar to control (Fig. 1f, g). Taken together, these findings suggest that HSV-MSLN effectively delivers MSLN to infected cells, induces the lysis of pancreatic cancer cells, releasing mediators that promote DCs maturation and potentially shift the TME to a more immunogenically active status.

### HSV-MSLN safely suppresses Pan02 tumor growth accompanied by an increase in the infiltrated CD8^+^ T cells

**Fig. 2:**
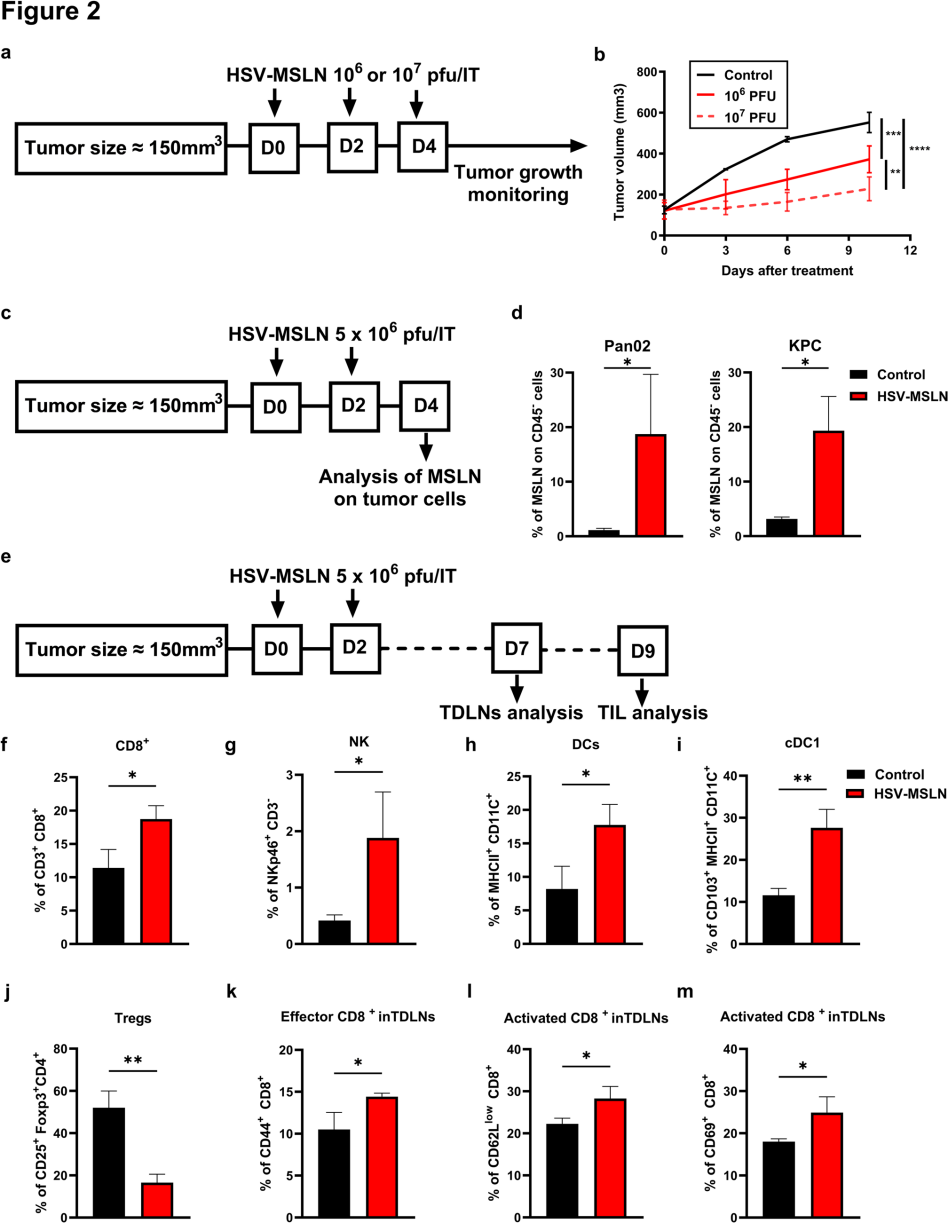
HSV-MSLN exhibits an antitumor effect in PDAC tumor-bearing mice. **a** A scheme shows the schedule of HSV-MSLN treatment in C57BL/6 Pan02 tumor-bearing mice. Mice were subcutaneously inoculated with Pan02 tumors, and before the treatment, mice were randomly divided into three groups (*n* = 3 mice per group) with an equal average tumor volume among the groups. HSV-MSLN was injected at either 1 × 10^6^ pfu or 1 × 10^7^ pfu three times IT every other day. Tumor sizes were measured twice a week. **b** Representative tumor growth in Pan02 tumor models after treatment. Data are presented as mean ± SD. Two-Way ANOVA followed by Tukey’s multiple comparisons tests were performed. **p < 0.01, ***p < 0.001, ****p < 0.0001. **c** A scheme shows the schedule of HSV-MSLN treatment in Pan02 and KPC tumor-bearing mice. Mice were subcutaneously inoculated with tumors. HSV-MSLN was IT injected at 5 × 10^6^ PFU twice. On day 4, tumors were digested into single cells and stained with anti-CD45 and anti-MSLN antibodies. **d** Percentage of CD45^−^ MSLN^+^ cells is displayed in the bar graph. Data are presented as mean ± SD. A student’s t-test was performed. *p < 0.05. **e** A scheme shows the schedule of HSV-MSLN treatment in subcutaneous Pan02 tumor. HSV-MSLN was IT injected at 5 × 10^6^ PFU twice. **f–j** On day 9, tumors were digested into single cells and stained with the indicated antibodies. Percentages of each population in each group are displayed in bar graphs **f** CD3^+^CD8^+^; **g** NKp46^+^CD3^−^; **h** DCs (CD11c^+^ MHCII^+^); **i** cDC1 (CD103^+^ CD11c^+^ MHCII^+^); and **j** Tregs (CD25^+^ Foxp3^+^ CD4^+^). **K–m** TDLNs were harvested on day 7 in **f**–**j**. Cells were stained with indicated antibodies and analyzed by flow cytometer. Bar graphs showing the percentages of CD44^+^ in CD8^+^ T cells (**k**), CD62L^low^ T cells in CD8^+^ cells (**l**), or CD69^+^ in CD8^+^ T cells (**m**). Data are presented as mean ± SD. A student’s t-test was performed. *p < 0.05, **p < 0.01

Next, we examined whether HSV-MSLN can function as an oncolytic virus in vivo since HSV-MSLN is derived from the clinical wild-type HSV1, which might have substantial neurotoxicity [29]. First, to ensure safety, C57BL/6 mice were inoculated subcutaneously with Pan02 tumors. When tumor size reached approximately 150 mm^3^, a low (1 × 10^6^ pfu) or a high (1 × 10^7^ pfu) dose of HSV-MSLN was injected IT according to the scheme in Fig. 2a. The mice did not exhibit signs of clinical illness or weight loss, even in the high dose, indicating the safety of the HSV-MSLN (Fig. S2). We also briefly monitored tumor growth during the safety experiment. As expected, HSV-MSLN treatment was able to delay Pan02 tumor growth (Fig. 2b). To assess the MSLN delivery efficiency in vivo, PDAC tumor-bearing mice were treated twice IT with 5 × 10^6^ pfu HSV-MSLN (Fig. 2c). The MSLN expression on tumor cell surfaces was analyzed by flow cytometer. We found the MSLN expression on CD45-negative cells in the HSV-MSLN-treated group, reaching around 20% in both Pan02 and KPC tumor-bearing mice (Fig. 2d). These results indicate that HSV-MSLN exhibits safety and efficacy as an oncolytic virus with a high potential of delivering MSLN (CAR targets) to the tumor cell surface.

We further investigate the impact of HSV-MSLN on the immune cells in the TME. We analyzed the immune cells in Pan02 tumors after treatment with 5 × 10^6^ pfu HSV-MSLN (Fig. 2e). The gating strategy is shown in Fig. S3. We found that HSV-MSLN treatment significantly increased infiltrated CD3^+^ CD8^+^ T cells (Fig. 2f), which play a major role in the antitumor effect. We further observed a significant increase in NK population (NKp46^+^ CD3^−^) in HSV-MSLN-treated tumors compared to control (Fig. 2g). Regarding the innate immune cells, DCs (CD11c^+^ MHCII^+^) were significantly increased in the virus-treated group (Fig. 2h). Conventional DC (cDC1) plays an important role in attracting, proper activation, and maintenance of the effector function of CD8^+^ TILs [34]. Here, cDC1 subsets (CD103^+^ CD11c^+^ MHCII^+^) were significantly increased after HSV-MSLN treatment (Fig. 2i). Although we didn’t find a significant difference in the CD4^+^ population between the HSV-MSLN and the control groups (Fig. S4a), Tregs (CD4^+^ Foxp3^+^ CD25^+^) were dramatically decreased in the HSV-MSLN group compared to the control group (Fig. 2j). Additionally, both macrophages (CD11b^+^ F4/80^+^) and inflamed macrophages (ly6c^+^ CD11b^+^ F4/80^+^) had the tendency to increase in HSV-MSLN-treated tumors (Fig. S4b, c). These data suggested that HSV-MSLN treatment changed the TME toward a more immunologically active state.

To clarify the high infiltration of immune cells in the tumors, we collected tumor-draining lymph nodes (TDLNs) on day 7 after the HSV-MSLN treatment and analyzed DCs and CD8^+^ T cells populations. We found that DCs (CD11c^+^ MHCII^+^) significantly increased in the TDLNs of the HSV-MSLN-treated group (Fig. S4d). Although there was no significant difference in the percentage of CD8^+^ T cells between the control and HSV-MSLN-treated group (Fig. S4e), in the TDLNs of the HSV-MSLN-treated group, CD8^+^ T cells exhibited a significantly higher expression of the effector marker CD44 (Fig. 2k) and a significant increase in the activated CD62L^low^ CD8^+^ population (CD62L is a marker for naïve CD8^+^ T cells) (Fig. 2l). Additionally, the activation marker CD69 was more highly expressed in the CD8^+^ of the TDLNs of the HS-MSLN-treated group (Fig. 2m). This phenotypic shift implies that HSV-MSLN treatment may be promoting priming of naïve CD8^+^ T cells in TDLNs to become effectors and activated cells that infiltrate to the tumor site.

### MSLN delivered to the HSV-MSLN-infected cells activates MSLN-CAR T cells in vitro

To address whether the MSLN expression on the pancreatic cancer cells activates MSLN-targeting CAR T cells, we generated MSLN-CAR T cells by transducing murine splenic T cells with a retrovirus harboring second-generation CARs, which included an MSLN-targeting scFv, CD3ζ, CD28 co-stimulatory signaling molecules, and GFP (Fig. S5a). After the transduction of the CAR construct, GFP-positive T cells were analyzed by flow cytometer, revealing that nearly 70% of murine T cells were GFP-positive (Fig. S5b). To evaluate the reactivity of MSLN-CAR T cells to MSLN-expressing cells, MSLN-CAR T cells were co-cultured with MSLN-expressing Pan02 cells (Pan02-MSLN). Subsequently, the intracellular IFN-γ production was assessed. When co-cultured with Pan02-MSLN cells, around 63% of MSLN-CAR T cells were activated and produced high levels of IFN-γ, while neither MSLN-CAR T cells co-cultured with MSLN-non-expressing Pan02 cells nor untransduced mock-T cells co-cultured with Pan02-MSLN exhibited high levels of IFN-γ (Fig. S5c). Those results suggest that the generated MSLN-CAR T cells could be functionally activated in a MSLN-specific manner.

**Fig. 3:**
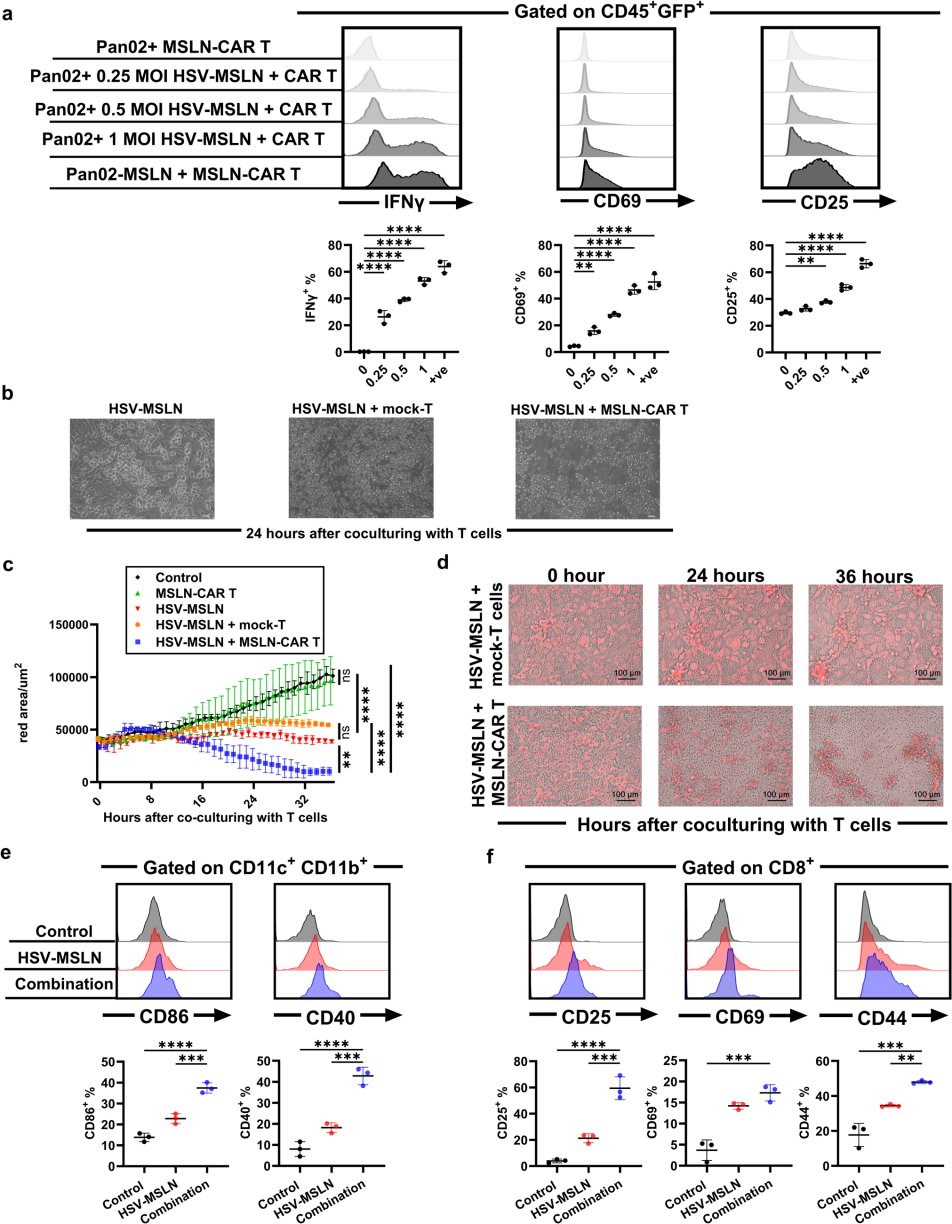
The delivery of MSLN by HSV-MSLN confers the activation and cytotoxicity of MSLN-CAR T cells against the infected cancer cells. **a** Top: representative histogram of flow analysis showing the abundance of intracellular IFN-γ (left), cell surface CD69 (middle), and CD25 (right) in MSLN-CAR T cells after 6-h co-culture with Pan02 cells or Pan02 cells infected with 0.25, 0.5, or 1 MOI of HSV-MSLN or Pan02-MSLN. The ratio of effector MSLN-CAR T cells to Pan02 cells was 1:1. Bottom: Summary of percentage of IFN-γ^+^ (left), CD69^+^ (middle), and CD25^+^ (right). Data are presented as mean ± SD (*n* = 3). One‐Way ANOVA followed by Dunnett’s multiple comparison tests were performed. **p < 0.01, ****p < 0.0001. **b** Representative images of Pan02 cells infected with 0.5 MOI of HSV-MSLN for 24 h, followed by the addition of media (left), mock non-transduced T cells (middle), or MSLN-CAR T cells (right). Bars are 50 µm. **c** Quantification of the red area to assess KPC-RFP cells killing using BZ-X800 analyzer software (Keyence). KPC-RFP cells were monitored over a 36-h period under the indicated conditions. Data are presented as mean ± SD (*n* = 3). Two-Way ANOVA followed by Tukey’s multiple comparison test was performed. **p < 0.01, ****p < 0.0001. **d** Representative images of KPC-RFP cell killing from time-lapse imaging at the indicated time point. Bars are 100 µm. KPC-OVA cells were infected with HSV-MSLN at an MOI of 0.5 for 18 h. The infected cells were co-cultured with or without MSLN-CAR T cells at E: T ratio of 1:1 for 20 h. The resulting culture supernatants were collected and used to condition immature BMDCs for 6 h. Naïve OT-1 CD8^+^ T cells were then added at a DC: T cell ratio of 1:4 and incubated for 36 h then subjected to flow cytometry. Three experimental conditions were included: Control (culture supernatant from non-infected KPC-OVA cells), HSV-MSLN (culture supernatant from HSV-MSLN-infected KPC-OVA cells), and Combination (culture supernatant from HSV-MSLN-infected KPC-OVA cells co-cultured with MSLN-CAR T cells). **e** Top**:** Representative histogram of flow analysis showing the abundance of cell surface CD86 (left) and CD40 (right) on BMDCs. Bottom: Summary of percentage of CD86^+^ (left), and CD40^+^ (right). Data are presented as mean ± SD (*n* = 3). **f** Top: Representative histogram of flow analysis showing the abundance of cell surface CD25 (left), CD69 (middle), and CD44 (right) on OT-1 CD8^+^ T cell. Bottom: Summary of percentage of CD25^+^ (left), CD69^+^ (middle), and CD44^+^ (right). Data are presented as mean ± SD (*n* = 3). One‐Way ANOVA followed by Dunnett’s multiple comparison tests were performed. **p < 0.01, ***p < 0.001, ****p < 0.0001

Next, we assessed the activity of MSLN-CAR T cells co-cultured with HSV-MSLN-infected pancreatic cancer cells. Pancreatic cancer cell lines (Pan02 and KPC) were infected with the HSV-MSLN virus at increasing MOIs for 24 h. MSLN-CAR T cells were subsequently co-cultured with the infected cells at a 1:1 ET ratio for 6 h and assessed with IFN-γ, CD69, and CD25 as activation markers on T cells. An increasing IFN-γ intracellular production, CD69, and CD25 surface expression in MSLN-CAR T cells were clearly observed after co-culturing with HSV-MSLN-infected Pan02 cells in a MOI-dependent manner (Fig. 3a). Similar observations for IFN-γ and CD69 were seen with the use of the KPC cells (Fig. S6a, b). These results indicate that MSLN-CAR T cells were activated by the MSLN delivered by the HSV-MSLN on the infected pancreatic cancer cells. Next, to check the cytotoxicity of MSLN-CAR T cells through the activation, the target cell (Pan02) death was assessed by phase-contrast microscopy. The infected Pan02 cells co-cultured with MSLN-CAR T cells were completely killed compared to the HSV-MSLN-infected cells not co-cultured with MSLN-CAR T cells or co-cultured with mock-T cells (Fig. 3b).

To assess the kinetics of the cytotoxicity of MSLN-CAR T cells, the fluorescent time-lapse imaging analysis was performed to monitor RFP-expressing KPC cells. KPC-RFP cells were infected or not infected with HSV-MSLN at a 0.5 MOI. After 24 h, MSLN-CAR T cells or mock-T cells were added, and cell death was monitored. Without HSV-MSLN infection, the total red area was gradually increased due to cells proliferation (Fig. 3c, control, Supplementary file. 2). Similar results were obtained in non-infected KPC-RFP cells co-cultured with MSLN-CAR T cells, suggesting MSLN-CAR T cells inactivity was due to the absence of target antigen (MSLN) (Fig. 3c, MSLN-CAR T, Supplementary file. 3). When KPC-RFP cells were infected with HSV-MSLN, regardless of the presence of mock-T cells, the total red area was almost sustained (Fig. 3c, HSV-MSLN and HSV-MSLN + mock-T, Supplementary file. 4, 5). In contrast, when the HSV-MSLN-infected cells were co-cultured with MSLN-CAR T cells, the total red area was clearly decreased, strongly indicating the superior cytotoxicity and the specificity of MSLN-CAR T cells (Fig. 3c, d, Supplementary file. 6, 7). These results suggest that HSV-MSLN delivers MSLN to cancer cells, allowing MSLN-CAR T cells to target and kill them more effectively, thereby enhancing HSV-MSLN-mediated tumor cell lysis.

To determine whether this enhanced tumor cell lysis also promotes DC maturation, antigen presentation, and T cell priming, we conducted an in vitro co-culture experiment. Immature BMDCs were conditioned with supernatants from KPC-OVA cells infected with HSV-MSLN, either alone or in combination with MSLN-CAR T cells. These conditioned BMDCs were then co-cultured with naïve OT-1 CD8^+^ T cells. BMDCs conditioned with the combination of HSV-MSLN and MSLN-CAR T cells exhibited significantly increased expression of CD86 and CD40, indicating enhanced DC maturation (Fig. 3e) compared to those conditioned with HSV-MSLN alone. This enhanced maturation can be attributed to the enhanced tumor cell lysis induced by both HSV-MSLN and MSLN-CAR T cells, which release inflammatory mediators that synergize with the virus-derived mediators to significantly enhance DC maturation and activation. Additionally, the increased tumor cell lysis in the combination group leads to more OVA antigen release from KPC-OVA cells, which is taken up by DCs, processed, and cross-presented to naïve OT-1 T cells, thereby mediating their priming and activation. Here we observed that, naïve OT-1 T cells co-cultured with these conditioned BMDCs exhibited increased expression of CD25, CD44, and CD69 (Fig. 3f) in the combination group compared to HSV-MSLN alone, suggesting improved antigen presentation and T cell priming.

### The combination of HSV-MSLN with MSLN-CAR T cells enhances antitumor activity in PDAC tumor-bearing mice

**Fig. 4:**
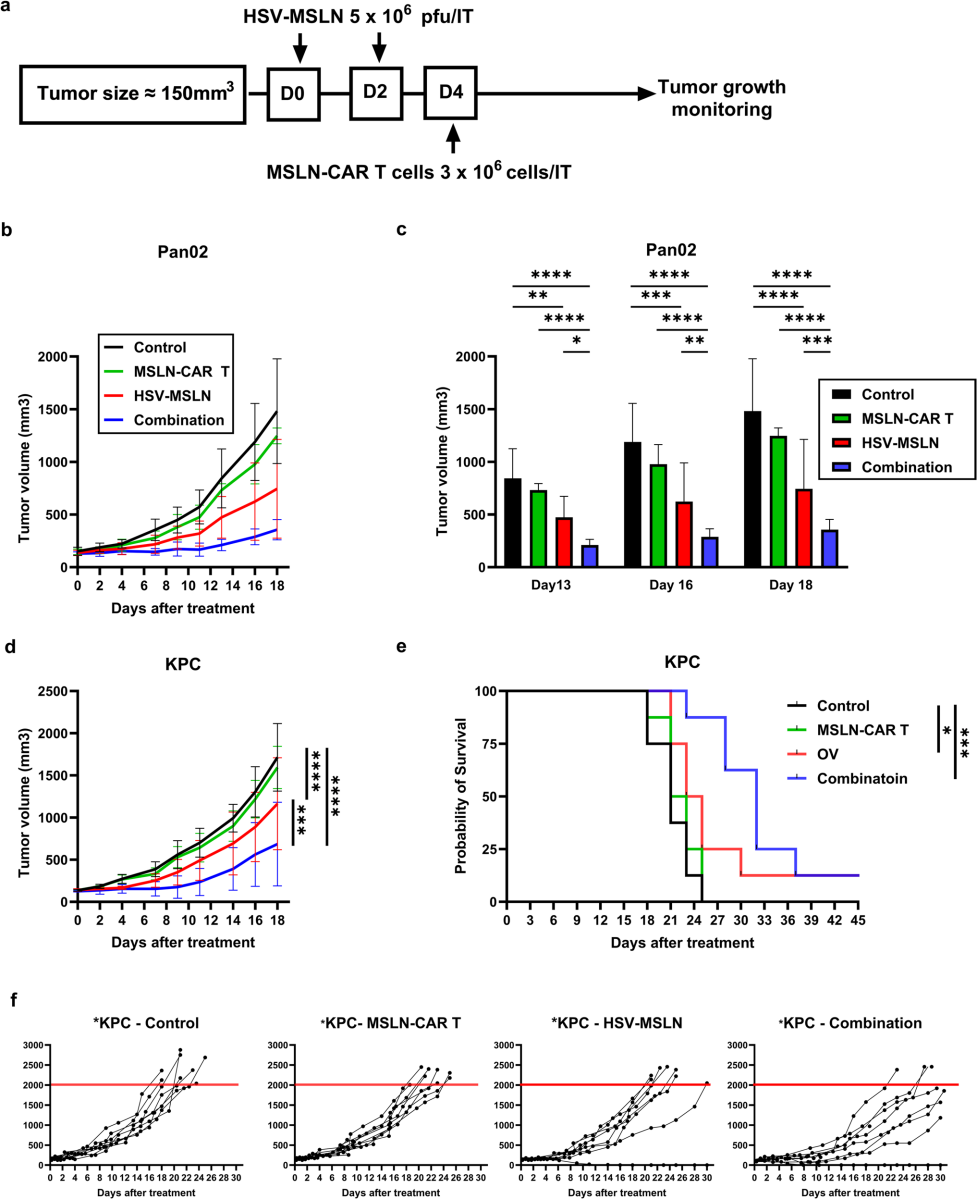
HSV-MSLN enhances the antitumor effect of MSLN-CAR T cells in PDAC tumor-bearing mice. **a** A scheme shows the treatment schedule for PDAC tumor-bearing mice. Mice were randomly divided into four groups with equal average tumor volumes, around 150 mm^3^, among the groups. HSV-MSLN was injected twice IT at 5 × 10^6^ PFU (D0, D2). On day 4, MSLN-CAR T cells were injected IT at a dose of 3 × 10^6^ cells. Tumor sizes were measured 3 times/ week. **b** Tumor growth curves of treated Pan02 tumors (*n* = 6 mice/group). **c** Tumor size data from (**b**) on Day 13, 16, and 18. Two-Way ANOVA followed by Tukey’s multiple comparisons tests were performed. *p < 0.05, **p < 0.01, ***p < 0.001, ****p < 0.0001. **d** Tumor growth curve of KPC tumors (*n* = 8/group) until day 18. Data are presented as mean ± SEM. Two-Way ANOVA followed by Tukey’s multiple comparisons tests were performed. ***p < 0.001, ****p < 0.0001. **e** Kaplan–Meier curve from (**d**). For the evaluation of survival, the death event was defined when the total tumor size reached 2000 mm^3^. The log-rank test was used for statistical comparison. *p < 0.05, ***p < 0.001. **f** Individual tumor growth curves from (**d**). Tumors reaching 2000 mm^3^ are marked with red lines

In vitro, MSLN-CAR T cells are activated and efficiently kill infected cells through delivered MSLN. Therefore, we evaluated the combination treatment of antigen-delivering oncolytic virus (HSV-MSLN) with MSLN-CAR T cells in vivo using the Pan02 subcutaneous tumor-bearing mice model. When the tumor size reached around 150 mm^3^, treatment started according to the scheme in Fig. 4a. MSLN-CAR T cells monotherapy didn’t suppress tumor growth and showed tumor growth comparable to the control, with no significant difference at any time point (Fig. 4b, MSLN-CAR T). This result was expected due to the absence of MSLN expression in the tumor cells, which is the target of the injected MSLN-CAR T cells. Consistent with Fig. 2c, HSV-MSLN monotherapy showed significant antitumor activity and slowed tumor growth compared to the control and the MSLN-CAR T cells groups (Fig. 4b, HSV-MSLN). The suppression in the HSV-MSLN group is likely due to the modulation of the TME by HSV-MSLN, as shown in Fig. 2f–m. Interestingly, combination therapy showed significant antitumor activity compared to HSV-MSLN monotherapy on days 13, 16, and 18 (Fig. 4b, Combination, c). We further repeated this treatment scheme (Fig. 4a) in KPC tumor-bearing mice. Similar to the results of the Pan02 tumor model, both the HSV-MSLN group and the combination group significantly inhibited tumor growth compared to the control and MSLN-CAR T cell groups (Fig. 4d, f). Notably, the combination group exhibited stronger inhibitory effects than HSV-MSLN monotherapy (*P* < 0.001). Moreover, the combination group showed synergism from the calculation of the synergistic effect on both the Pan02 and KPC tumors (Table.S2). In KPC tumor-bearing mice, we further analyzed 45-day survival in all groups. Along with the animal guidelines, mice were sacrificed when the tumor size exceeded the volume of 2000 mm^3^. In the control group, mice began to succumb on day 18, with 100% mortality by day 25. A similar trend was observed in the MSLN-CAR T cells group (Fig. 4e, f, Control, and MSLN-CAR T). Compared to the control group, the HSV-MSLN group exhibited prolonged overall survival with complete tumor regression (CR) in one mouse (*P* < 0.05) (Fig. 4e, f, HSV-MSLN). The combination group demonstrated significantly prolonged overall survival compared to the control and MSLN-CAR T cells groups, as 7 out of 8 mice survived until day 25, including CR in one mouse (*P* < 0.001) (Fig. 4e, f, Combination). Additionally, all groups exhibited a steady increase in body weight, and no significant signs of toxicity were observed throughout the study, confirming the safety of HSV-MSLN treatment (Fig. S7).

### HSV-MSLN reprograms the TME enhancing MSLN-CAR T cells function

**Fig. 5:**
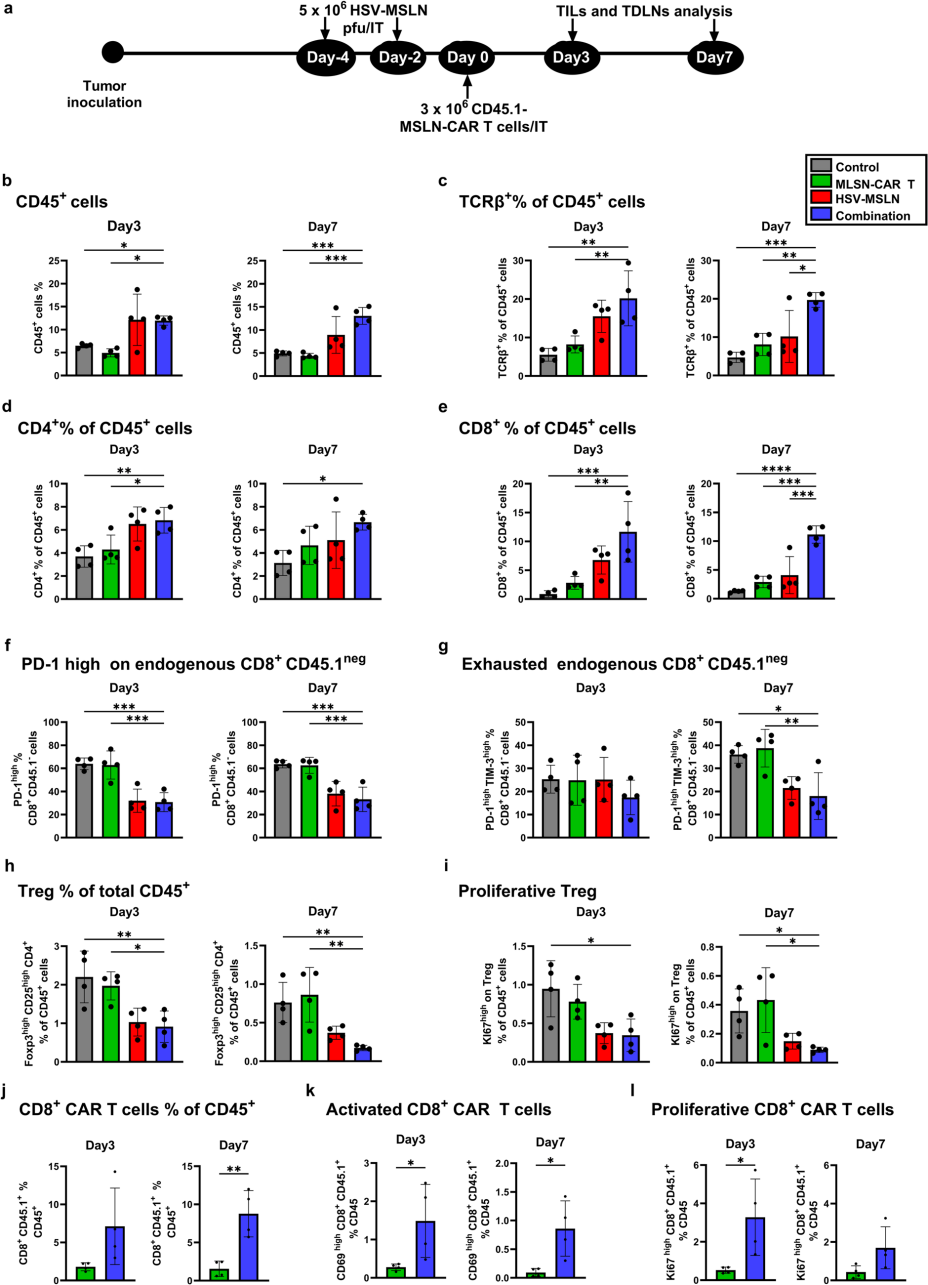
HSV-MSLN reprograms the TME enhancing MSLN-CAR T cell function. **a** A scheme shows the treatment schedule for tumor-bearing mice. C57BL/6J/CD45.2 mice (*n* = 4) were subcutaneously implanted with KPC tumors and treated with two doses of HSV-MSLN (5 × 10⁶ PFU), followed by a single dose of MSLN-CAR T cells (3 × 10⁶ cells). The MSLN-CAR T cells were prepared from the spleens of C57BL/6J/CD45.1 mice to enable distinction between endogenous and transferred T cells. **b–l** A single-cell suspension was prepared from tumor tissue 3 and 7 days after the last treatment and stained with the indicated antibodies. Percentages of each population in each group are displayed in bar graphs **b** CD45^+^; **c** TCRβ^+^ % of CD45^+^; **d** CD4^+^ % of CD45^+^; **e** CD8^+^ % of CD45^+^; **f** PD-1 expression level on endogenous CD8^+^ CD45.1^−^; **g** TIM-3 and PD-1 co-expression level on endogenous CD8^+^ CD45.1^−^; **h** Tregs (Foxp3^+^ CD25^+^) on endogenous CD4^+^ CD45.1^−^ % of CD45^+^; **i** Ki67 expression level on Treg % of CD45^+^; **j** transferred CD45.1^+^ CD8^+^ CAR T cells % of CD45^+^; **k** CD69^+^ CD8^+^ CD45.1^+^ % of CD45^+^ cells; and **l** Ki67^+^ CD8^+^ CD45.1^+^ % of CD45^+^ cells. Statistical analysis was performed using one-way ANOVA followed by Dunnett’s multiple comparison test for comparisons among more than two groups, and Student’s *t*-test for comparisons between two groups. All values are presented as the mean ± SEM. *p < 0.05, **p < 0.01, ***p < 0.001, and ****p < 0.0001

Our results in the murine tumor model demonstrated that combination therapy exhibited the greatest antitumor effects and prolonged survival compared to control, MSLN-CAR T cells, and HSV-MSLN monotherapies (Fig. 4). To investigate the changes in the TME, KPC tumors treated with each therapy were subjected to TILs analysis, following the schedule outlined in Fig. 5a. To distinguish endogenous lymphocytes from transferred CAR T cells, MSLN-CAR T cells were prepared from CD45.1 mice. Tumor tissues were harvested on day 3 and day 7 after the end of the treatment, and TILs were analyzed by flow cytometer (gating strategy shown in Fig. S8a).

Compared to the control and MSLN-CAR T cells monotherapy, the percentages of total CD45^+^ cells increased in HSV-MSLN monotherapy and combination therapy at day 3 and the effects were sustained until day 7 in combination therapy (Fig. 5b). This trend is similarly observed in the population of TCRβ⁺ T cells, including CD4⁺ T cells and CD8⁺ T cells (Fig. 5c–e). Furthermore, the combination therapy showed a significant increase in total TCRβ⁺ T cells and CD8^+^ T cells compared to the HSV-MSLN monotherapy at day 7 (Fig. 5c, e, Day7). Interestingly, analysis of endogenous T cells (CD45.1^neg^) showed a comparable increased number of CD4⁺ T cells and CD8⁺ T cells in HSV-MSLN monotherapy and combination therapy both at day 3 and day 7 compared to those in control and MSLN-CAR T cell monotherapy (Fig. S8b, c). Moreover, activated CD69^high^ endogenous CD8^+^ T cells increased in combination and HSV-MSLN group at day 3 but is comparable in all groups at day 7 (Fig. S8i). In addition, the percentages of Ki67 positive CD4^+^ and CD8^+^ cells, which are proliferative, were almost comparable among all groups both at day 3 and day 7 (Fig. S8d, e), indicating virus-mediated influx of endogenous T cells in HSV-MSLN monotherapy and combination therapy. We further examined the development of T cells exhaustion. In control, at day 3 approximately 60% of CD8^+^ T cells expressed PD-1 highly, but around 40% of them co-expressed TIM-3 highly (Fig. 5f, g, Day3). At day 7, PD-1^high^TIM-3^high^ cells reached around 60% of PD-1^high^ cells (Fig. 5f, g, Day7), indicating CD8^+^ T cells gradually became terminally exhausted status. However, those newly infiltrated endogenous CD8^+^ T cells in HSV-MSLN monotherapy and combination therapy remained unexhausted at day 7, while control and CAR T monotherapy exhibited an increasing number of PD-1^high^TIM-3^high^ exhausted CD8^+^ T cells (Fig. 5f, g), suggesting that the TME in HSV-MSLN monotherapy and combination therapy remained less immunosuppressive. Indeed, the numbers of Treg cells significantly decreased with less expression of Ki67 in HSV-MSLN monotherapy and combination therapy both at day 3 and day 7 (Fig. 5h, i).

To further explore the dynamics of CAR T cells, CD45.1^pos^ CAR T cells were analyzed between the combination group and the CAR T cell monotherapy group. The exogenous CD45.1^pos^ cells were expanded more in the combination therapy at both time points (Fig. S8f). Similarly, CD4⁺ CAR T cells or CD8⁺ CAR T cells in the combination therapy exhibited an expansion at both time points (Fig. 5j, S8g). These sustained expanded CD8^+^ CAR T cells also displayed higher activation marker CD69 (Fig. 5k). The percentages of Ki67^+^ CD8⁺ CAR T cells were significantly higher in the combination at day 3, but not at day 7 (Fig. 5l), indicating that virus-delivered MSLN antigen activated MSLN-CAR T cells at the early timepoint and cleared the infected cells rapidly. In addition, similar to the endogenous CD8^+^ T cells, those expanded CD8^+^ CAR T cells remained relatively unexhausted (Fig. S8h). Taken together, viral treatment changed the TME into less immunosuppressive, allowing activated CAR T cells to proliferate with less exhaustion.

### Combination therapy enhances DC activity and T cell response in tumor and TDLN

To elucidate how the combination therapy modulates antigen-presenting cells (APC), we analyzed macrophages and DCs populations in the tumor at days 3 and 7 post-treatment. In the HSV-MSLN monotherapy, pro-inflammatory macrophages (MHCII^high^ Ly6C^high^) increased the most at day 3 (Fig. S9a, b, Day3), while the combination therapy also exhibited a significant increase in MHCII^high^ Ly6C^high^ macrophages at day 3 compared to control and MSLN-CAR T cell monotherapy (Fig. S9a, b, Day3). By day 7, MHCII^high^ Ly6C^high^ macrophages remained elevated in both the HSV-MSLN and combination groups compared to control and MSLN-CAR T cells (Fig. S9b, Day7). Conversely, MHCII^low^ Ly6C^low^ macrophages, associated with immunosuppressive functions, were significantly decreased in HSV-MSLN monotherapy and combination therapy compared to control and MSLN-CAR T monotherapy at both time points (Fig. S9a, c).

**Fig. 6:**
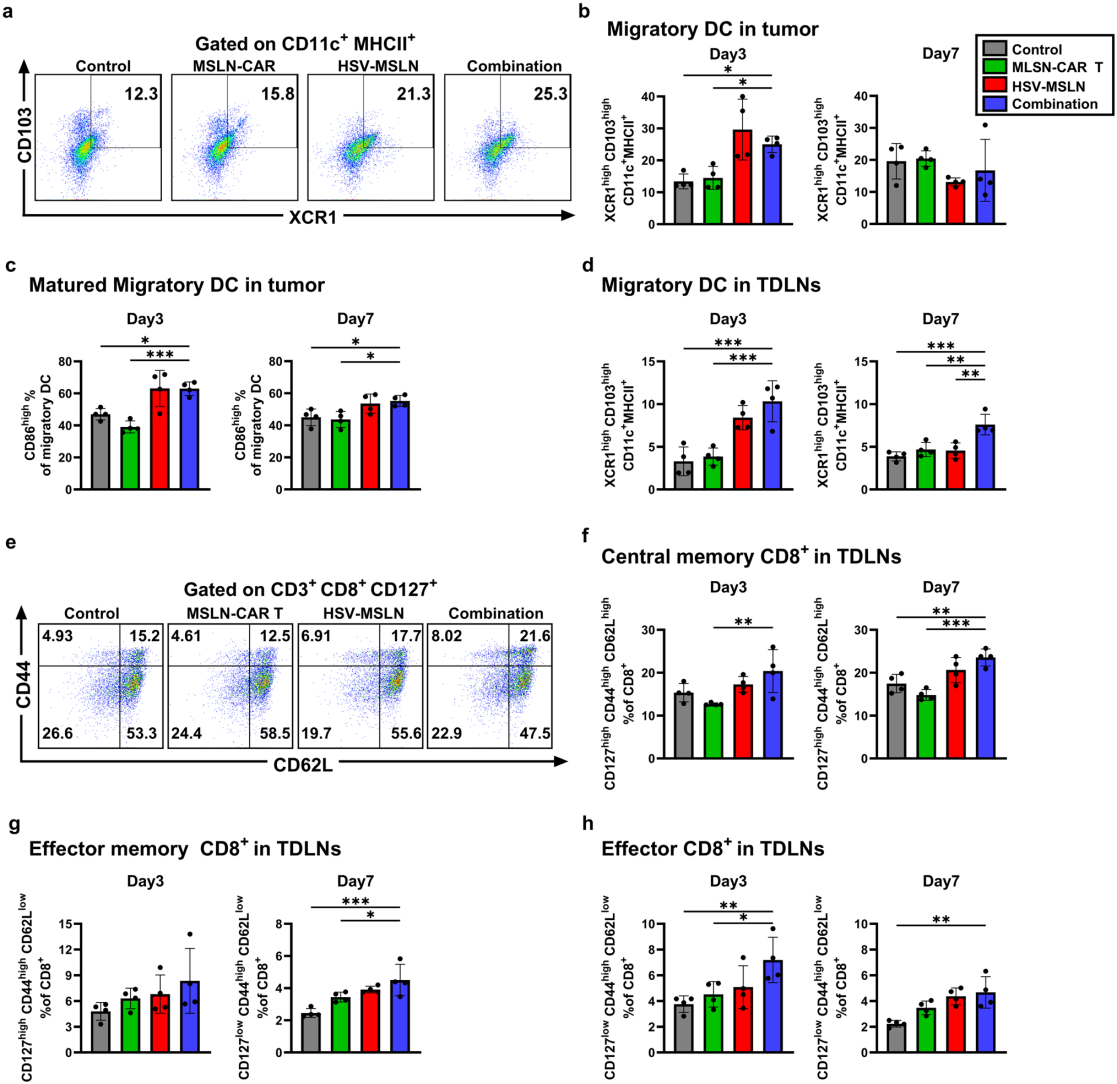
Combination therapy enhances DCs activity and T cell response in tumor and TDLN. Mice (*n* = 4) were subcutaneously implanted with KPC tumors and treated with two doses of HSV-MSLN (5 × 10⁶ PFU), followed by a single dose of MSLN-CAR T cells (3 × 10⁶ cells). A single-cell suspension was prepared from tumor tissue 3 and 7 days after the last treatment and stained with indicated antibodies. **a** Representative dot plot of XCR1^high^ CD103^high^ gated on CD45^+^ MHCII^+^ CD11c^+^ cells from the four treated groups at day 3. **b** Bar graphs show percentages of migratory DCs (XCR1^high^ CD103^high^) in tumor tissue at day 3. **c** Bar graphs show percentage of matured CD86^high^ migratory DCs in tumor. TDLNs were harvested, digested and stained with indicated antibodies. **d** Bar graphs show percentages of XCR1^high^ CD103^high^ on CD11c^+^ MHCII^+^ in TDLNs. **e** Representative dot plot of CD62L^high^ CD44^high^ gated on CD3^+^ CD8^+^ CD127^high^ from the four treated groups in TDLNs at day 3. **f–h** Percentages of each population in each group are displayed in bar graphs **f** CD62L^high^ CD44^high^ CD127^high^ CD8⁺ T cells; **g** CD62L^low^ CD44^high^ CD127^high^ CD8⁺ T cells; and **h** CD62L^low^ CD44^high^ CD127^low^ CD8⁺ T cells. Statistical analysis was performed using one-way ANOVA followed by Dunnett’s multiple comparison test. All values are presented as the mean ± SEM. *p < 0.05, **p < 0.01 and ***p < 0.001

Aside from macrophages, HSV-MSLN monotherapy and combination therapy significantly increased migratory DCs (XCR1^high^ CD103^high^) in the tumor compared to control and MSLN-CAR T cells groups at day 3, which was not observed at day 7 (Fig. 6a, b). Similarly, mature migratory DCs expressing CD86 were increased in HSV-MSLN monotherapy and combination therapy (Fig. 6c). Since by day 7, all groups showed comparable levels of migratory DCs, we hypothesized that DCs migrated to the TDLNs to present antigens.

In the TDLNs, HSV-MSLN monotherapy and combination therapy led to a significant increase in migratory XCR1^high^ CD103^high^ DCs at day 3 compared to control and MSLN-CAR T cells (Fig. 6d). Notably, at day 7, the percentages of XCR1^high^ CD103^high^ DCs in the combination group were significantly higher compared to all other groups, suggesting that the combination treatment leads to a sustained increased cross-presenting DCs population in the TDLNs (Fig. 6d). Moreover, mature migratory DC increased in combination and HSV-MSLN group in both days (Fig. S9e). Along with those observations, combination therapy enhanced the percentage of CD44^+^ CD8⁺ T cells both at days 3 and 7 in TDLNs (Fig. S9d). We further investigated the memory compartment among CD44^+^ CD8⁺ T cells in TDLNs. Combination therapy exhibited the highest percentage of central memory CD62L^high^ CD44^high^ CD127^high^ CD8⁺ T cells (TCM), effector memory CD62L^low^ CD44^high^ CD127^high^ CD8⁺ T cells (TEM), and effector CD62L^low^ CD44^high^ CD127^low^ CD8⁺ T cells, respectively (Fig. 6e–g).

## Discussion and conclusion

We chose MSLN, a tumor antigen, as a CAR target for pancreatic cancer due to its promising expression profile in the body. Indeed, several clinical trials were conducted against MSLN as a therapeutic target despite showing marginal benefits. For example, in a phase I clinical trial (NCT01897415) using MSLN-CAR T cells for pancreatic cancer, stable disease was observed in only 2 out of 6 patients [35]. Another phase I trial (NCT02159716) showed no remarkable clinical responses of MSLN-CAR T cells in various solid cancers, including pancreatic cancer. This was later explained by findings that MSLN expression on tumor cells was > 75% in only 3 out of 15 patients [36]. The heterogeneous MSLN expression has been reported in various solid tumors, with some tumors showing lower or no expression despite previously reported to be positive, including pancreatic cancers [19]. One explanation for the expression loss of MSLN is that MSLN undergoes routine extracellular shedding, leading to its downregulation of tumor cells [37]. This shedding is mediated by several proteases, with TACE/ADAM17 being the most notable [38]. The shedding of MSLN reduces the efficacy of various targeted therapies, including immunotoxins and CAR T cells; however, inhibiting this shedding using drugs that restrict the activity of these proteases has been shown to enhance the efficacy of MSLN-targeted therapies by increasing the presence of MSLN on the cell surface [39]. While the former approach focuses on preventing MSLN shedding, we explore the potential of increasing MSLN expression on the surface of tumor cells using OVs. Here, for the first time we engineered an oHSV1-expressing human MSLN (HSV-MSLN) and evaluated the combination therapy of HSV-MSLN and MSLN-CAR T cells in a murine PDAC model. HSV-MSLN, which is designed to increase MSLN expression on tumor cells. This approach would be beneficial in the clinical setting since, even though HSV-MSLN infected cells will eventually undergo oncolysis, the virus significantly boosts MSLN expression on their surface before cell death. This increase in antigen density, with both naturally expressed and virus-induced MSLN present, is critical for effective CAR T cell activation, as it improves recognition and signaling. Additionally, tumor cells that naturally express MSLN but are not infected by the virus will be targeted and killed by the more activated CAR T cells.

Aside from addressing the issue of MSLN expression loss, the combination of CAR T cells with OVs has gained significant attention. Researchers have suggested that the limited benefits of CAR T cells in solid tumors, due to the immunosuppressive TME and challenges in CAR T cell trafficking, can be overcome by genetically modified OVs [40]. Indeed, we and others demonstrated that intratumoral injection of oHSV leads to increased infiltration of T cells within the TME compared to non-treated groups [41–44]. Combining HSV-MSLN with MSLN-CAR T cells showed increased in both CD4^+^ and CD8^+^ T cells compared to HSV-MSLN monotherapy which is most likely attributed to the influx of endogenous T cells and the expansion of injected CAR T cells. This is particularly relevant in PDAC, which has a highly immunosuppressive TME that hinders CD8^+^ T cells infiltration and promotes the rapid induction of exhaustion markers such as PD-1 and TIM-3 on these cells [45]. We have previously reported that HSV-based OV decreased PD-1^high^ CD8^+^ T cells and PD-1^high^ TIM-3^high^ CD8^+^ T cells in TME [42, 44]. Consistent with our previous results, our TIL analysis revealed that HSV-MSLN decreases the PD-1^high^ CD8^+^ on both day 3 and day 7 and PD-1^high^ and TIM-3^high^ CD8^+^ T cells in TME especially at day 7. The decrease in exhausted CD8^+^ T cells is coupled with a significant decrease in Tregs in combination and HSV-MSLN treated groups. Previous studies have shown that oHSV1 armed with either IL-12 or GM-CSF reduced Tregs in sarcoma and melanoma [46, 47]. Here, we found that HSV-MSLN decreases Tregs levels without cytokine arming, similar to the findings previously reported in a murine PDAC tumor model [27].

The importance of APCs, especially migratory DCs, in orchestrating antitumor immunity has been clearly demonstrated in previous studies [48]. Both HSV-MSLN and the combination therapy increased migratory DCs (CD103^high^ XCR1^high^ CD11c⁺ MHCII⁺) and matured migratory DCs in the TME. This increase is likely driven by OV-induced tumor cell lysis, which releases tumor and viral antigens. These antigens are loaded onto DCs, which migrate to the TDLNs, prime naïve CD8⁺ T cells, and promote their infiltration into the TME. This is supported by our observation of an increase in migratory DCs as well as activated CD44^+^ CD8^+^ T cells infiltrating the TDLNs. We observed that the combination group exhibited the highest percentage of CD103^high^ XCR1^high^ cross-presenting migratory DCs at both day 3 and day 7, along with the highest percentage of primed CD44⁺ CD8⁺ T cells in TDLNs. Taken together, these findings suggest that the combination of CAR T cells and HSV-MSLN may enhance DC activation in TDLNs and promote the priming and activation of naïve T cells. However, this effect seems to be primarily driven by the HSV-MSLN, as the differences in immune cell subsets between the combination and HSV-MSLN groups were not consistently significant, despite showing elevated levels in the combination group. Future studies focusing on this aspect could help clarify how CAR T cells may augment the OV effect on DC function and improve the expansion of tumor-specific T cells, ultimately enhancing the overall efficacy of combination therapies.

Memory T cells, which are crucial for sustaining antitumor immunity [49], were also enhanced with combination therapy leading to an increase of CD62L^high^ CD44^high^ CD127^high^ CD8⁺ TCM, CD62L^low^ CD44^high^ CD127^high^ CD8⁺ TEM in TDLNs, highlighting enhanced survival and persistence of these cells. These observations suggest that combination therapy not only supports the activation and migration of antigen-presenting cells but also fosters the generation of functional CD8⁺ memory T cells subsets in the TDLNs.

Recent studies have investigated combining MSLN-CAR T cells with armed OVs for enhanced antitumor efficacy. MSLN-CAR T cells combined with an oncolytic adenovirus expressing IL-2 and TNF-α improved T cells function and CAR T cells tumor infiltration in pancreatic cancer models, enhancing antitumor efficacy and survival [50]. Another study found that combining MSLN-CAR T cells with an oncolytic adenovirus targeting TGF-ß improved antitumor activity in triple-negative breast cancer models [51]. Additionally, using an oncolytic vaccinia virus expressing C-X-C motif chemokine-11 (CXCL-11) with MSLN-CAR T cells enhanced CAR T cells tumor infiltration and antitumor activity in a lung cancer model [52]. This was also translated to clinical trials; in a phase I trial, combining VCN-01 (an oncolytic adenovirus expressing hyaluronidase) with anti-MSLN-CAR T cells showed feasibility and safety, with two ovarian cancer patients exhibiting stable disease for up to 150 and 300 days [53]. In these trends, enhancing the effect of CAR T cell therapy in solid tumors by increasing the level of target expression using OVs is a novel strategy. However, most studies employed CD19 or BCMA in their proof-of-concept because these CARs are among the most advanced clinically and FDA-approved for hematological malignancies [54–58]. However, CD19 and BCMA are also expressed in normal B cells, which can lead to unnecessary B-cell aplasia. To overcome this and explore the possibility of this strategy in the clinical setting, we chose MSLN as a CAR target. MSLN is naturally expressed in PDAC, and OVs can boost its expression, generating high antigen density for full activation of MSLN-CAR T cells.

We engineered HSV-MSLN from the clinical wild-type HSV1 strain by replacing both copies of the γ34.5 gene with the human MSLN gene-expressing cassette to reduce neurotoxicity and promote selective replication in tumor cells [29]. The γ34.5 genes are deleted in several generations of oHSV, including HSV1716, R3616 NV1020, G207, and the two approved G47∆ and T-VEC [28]. Engineered HSV-MSLN successfully induces MSLN expression in pancreatic cancer tumor cells in vitro, with up to 80% of cells expressing MSLN. Additionally, the viability of infected pancreatic cells decreases with increasing MOI over time. In vivo*,* the HSV-MSLN virus shows a significant antitumor effect when intratumorally injected into PDAC tumor-bearing mice with no signs of neurotoxicity and safety confirmation. However, the intratumoral administration of HSV-MSLN achieves successful MSLN expression in approximately 20% of tumor cells in syngeneic mice in vivo. Similar findings were reported when an oncolytic vaccinia virus armed with CD19 was injected into orthotopic B16-melanoma tumors, with only 24% of tumor cells expressed CD19 [56]. Despite the observed percentage, our combination therapy of HSV-MSLN virus with MSLN-CAR T cells has demonstrated significant antitumor effects in two pancreatic tumor models (Pan02 and KPC). This suggests that the expressed MSLN is sufficient to elicit a therapeutic response. For patients with pancreatic cancer, adjusting the virus dosage and the duration between shots, as well as administering booster shots of the virus, can increase MSLN expression and activate tumor-resident CAR T cells. Additionally, preconditioning the patients with radiation or chemotherapy, such as cyclophosphamide [56]. These treatments can suppress immunity, allowing for better viral spread and delayed virus clearance. This approach is particularly relevant since CAR T cell therapy in clinical settings is typically preceded by a chemotherapeutic regimen to deplete endogenous T cells and facilitate the expansion of exogenous T cells [59].

Considering that in our tumor models, none of the tumor cells expressed human MSLN. Thus, theoretically, in both HSV-MSLN-treated and combination groups, the primary target cells were those infected with HSV-MSLN, making the number of targetable cells comparable between the two groups. Nevertheless, the combination group clearly showed stronger and prolonged antitumor effects. This enhanced antitumor effect in the combination group could be attributed to the synergistic interaction between effects from HSV-MSLN and MSLN-CAR T cells. Indeed, the synergism calculation in Table. S2 suggests that combination therapy conferred more than additive effects. Besides the upregulation of MSLN expression, HSV-MSLN reprogrammed the TME to a pro-inflammatory status, recruiting immune cells and creating a more favorable condition for the activation and the expansion of the MSLN-CAR T cells. Indeed, MSLN-CAR T cells were proliferative as Ki67^high^, activated as CD69^high^, and unexhausted as TIM-3^low^ PD-1^low^. Ki67 upregulation of CD8^+^ CAR T cells in the combination group increased at day 3 but declined at day 7. This indicates that virus-delivered MSLN might be cleared before day 7, suggesting that the expanded CD8^+^ CAR T cells might become bystander cells at an early time point because no targetable cells existed. Interestingly, CD4^+^ CAR T cells were similarly expanded. Taken together, we speculate that those activated and expanded CD8^+^ CAR T cells and CD4^+^ CAR T cells secreted cytokines and chemokines to support the tumor immunity that was evoked by the OV, which conferred the synergistic anti-tumor effects in combination therapy.

Although our data strongly supports the efficacy of HSV-MSLN combined with MSLN-CAR T cells, certain limitations should be acknowledged. The absence of an oHSV1Δ34.5 virus not expressing MSLN in the in vivo experiments limits our ability to fully distinguish the specific antigen-delivery effects of HSV-MSLN from the broader immune-stimulatory properties of HSV-MSLN as an OV. Additionally, we didn’t carry out the in vivo experiment in tumors stably expressing human MSLN in syngeneic mice. Therefore, we couldn’t assess the heterogeneity of MSLN expression and the boosting effect of HSV-MSLN. However, our model can still be interpreted as the worst situation of complete loss of MSLN expression where HSV-MSLN can still induce sufficient expression of MSLN. To validate the broader applicability of our findings, further research is worth investigating in other tumors that express some level of MSLN. Another limitation lies in the use of a subcutaneous PDAC model instead of an orthotopic model. While orthotopic models better simulate the clinical setting, subcutaneous models are widely employed in initial preclinical studies due to their reproducibility and the ease of consistent tumor growth monitoring. Additionally, the subcutaneous model was more suitable for our HSV-MSLN injection schedule, as repeated HSV-MSLN administration in murine orthotopic tumor models poses significant challenges, making the subcutaneous model a practical and efficient choice for our study.

Currently, the preferred method of administration for CAR T cell therapy in blood cancers is intravenous (IV) injection, allowing circulating cancer cells to easily interact with injected CAR T cells and enabling CAR T cells to reach both primary and secondary metastatic sites. However, this method faces challenges in solid tumors due to poor CAR T cells trafficking to tumor sites. To address these issues, regional administration of CAR T cells is being explored in solid tumors, showing that fewer CAR T cells are needed to achieve tumor responses and potentially reduce systemic side effects [60]. Phase I trial (NCT02414269) demonstrated antitumor activity with regional administration of MSLN-CAR T cells for malignant pleural disease, with detectable CAR T cells in the peripheral blood of half the patients treated [61]. Additionally, some clinical trials (NCT02706782, NCT03267173) explored the regional administration of CAR T cells through vascular intervention in PDAC to increase their concentration at tumor sites. Therefore, our experiments adopt the local delivery approach of MSLN-CAR T cells. Moreover, researchers have explored the potential benefits of combining CAR T cells with OVs to enhance trafficking. OVs induce inflammation and stromal destruction, aiding CAR T cells recruitment. Furthermore, adding lymphocyte-attracting chemokines like CXCL-9, CXCL-10, CXCL-11, or CCL19 in CAR T cells or OVs genomes has shown promise [52, 62, 63]. If we modify HSV-MSLN to further express CXCL-10 chemokines in the future, it will enhance the CAR T cells effect in two ways: first, by promoting antigen expression, and second, by increasing trafficking of IV-administered CAR T cells to tumor sites.

In conclusion, we developed a novel combination therapy between MSLN-CAR T cells and oHSV1, which is genetically modified to deliver the MSLN-CAR target. Our combination strategy enhances the antitumor effect in a murine PDAC tumor model. On the one hand, HSV-MSLN reprograms the suppressive TME to become immunoactive. On the other hand, it delivers the MSLN to the tumor cells to activate MSLN-CAR T cells. Following these results, further efforts are needed to investigate the potential of combining HSV-MSLN with other MSLN-targeted therapies, such as immunotoxins and cancer vaccines. This approach could enhance treatment outcomes and amplify the impact of targeting MSLN in solid tumors.

## Supplementary Information

Below is the link to the electronic supplementary material.

Supplementary file1 (PDF 964 KB)

Supplementary file2 (MPEG 5752 KB)

Supplementary file3 (MPEG 8192 KB)

Supplementary file4 (MPEG 5110 KB)

Supplementary file5 (MPEG 6968 KB)

Supplementary file6 (MPEG 6766 KB)

Supplementary file7 (MPEG 137646 KB)

## Data Availability

The datasets generated and/or analyzed during the current study are available from the corresponding author on reasonable request.

## Abbreviations

ATC: Adoptive T cell
APCs: Antigen presenting cells
BCMA: B-cell maturation antigen
BiTEs: Bispecific T cells engagers
BMDCs: Bone marrow-derived DCs
CAR: Chimeric antigen receptor
cDC: Conventional dendritic cells
CR: Complete tumor regression
CXCL: C-X-C motif chemokine
DAMPs: Damage-associated molecular patterns
DCs: Dendritic cells
ET: Effector target
FDA: Food and Drug Administration
HSV-MSLN: Herpes simplex virus engineered to express human mesothelin
ICBs: Immune checkpoint blockades
IT: Intratumorally
IV: Intravenous
MOI: Multiplicity of infection
MSLN: Mesothelin
MTT: 2,5-Diphenyl-2H-tetrazolium bromide
NK: Natural killer
oHSV1: Oncolytic herpes virus type 1
OV: Oncolytic virus
Pan02-MSLN: Pan02 cells expressing MSLN
PAMPs: Pathogen-associated molecular patterns
PDAC: Pancreatic ductal adenocarcinoma
PFU/mL: Plaque-forming units per milliliter
q-PCR: Quantitative polymerase chain reaction
TAA: Tumor-associated antigen
TCM: Central memory T cell
TEM: Effector memory T cell
TILs: Tumor-infiltrating lymphocytes
TDLNs: Tumor-draining lymph nodes
TME: Tumor microenvironment
Tregs: T regulatory cells
T-VEC: Talimogene laherparepvec
