# Supplementary material for: Enhancing mesothelin CAR T cell therapy for pancreatic cancer with an oncolytic herpes virus boosting CAR target antigen expression
Source: Cancer Immunol Immunother. 2025 May 14;74(7):202. doi: 10.1007/s00262-025-04039-7 (PMC12078189; doi:10.1007/s00262-025-04039-7)
Supplement: Supplementary file 1 — Supplementary file1 (PDF 964 KB) [file 262_2025_4039_MOESM1_ESM.pdf]

**Cancer Immunology, Immunotherapy (submitted in 2024) – Mona Alhussein Aboalela et al. Supplementary Materials.**

**Supplementary Table 1**

| Gene | Probe | Froward primer | Reverse primer |
|---|---|---|---|
| US4 | 5′-/56-FAM/CGTCTGGAC/ZEN/ CAACCGCCACACAGGT/3IABkFQ/-3′ | GCAGGCACACG TAACGCACGCT | TTCTCGTTCCTCACTGCCTC CC |
| CMV promoter | 5′-/56-FAM/AGTACATCA/ZEN/ ATGGGCG TGGA /3IABKFQ/-3′ | CAATGGGGCGG AGTTGTTAC | ACCATGGTGATGCGGTTTTG |
| γ34.5 | 5′-/56-FAM/TCTCCGGA G/ ZEN/ AGACGATGG/3IABkFQ/-3′ | TCTAACGTTACA CCCGAGGC | GTATATATGCGCGGCTCCTG |

**Table.S1 Sequence of primers for qPCR.**

| Pan02 | | | | | | KPC | | | | | |
|---|---|---|---|---|---|---|---|---|---|---|---|
| **Days** | **FTV** | **FTV** | **E-FTV** | **O-FTV** | **E-FTV/O-FTV** | **Days** | **FTV** | **FTV** | **E-FTV** | **O-FTV** | **E-FTV/O-FTV[a]** |
| | HSV-MSLN | MSLN-CAR T | Combination | Combination | Combination | | HSV-MSLN | MSLN-CAR T | Combination | Combination | Combination |
| **0** | 0.950534253 | 1.01901185 | 0.968605668 | 0.926194427 | 1.045790861 | **0** | 1.021617661 | 1 | 1.021617661 | 0.972794724 | 1.050188324 |
| **2** | 0.855778583 | 0.85949244 | 0.735535222 | 0.715801574 | 1.027568602 | **2** | 0.837381779 | 1.002041233 | 0.83909107 | 0.743212901 | 1.129004985 |
| **4** | 0.790262172 | 0.941797753 | 0.744267138 | 0.688988764 | 1.080231169 | **4** | 0.614063216 | 0.978973889 | 0.601151855 | 0.567109482 | 1.060027866 |
| **7** | 0.614065583 | 0.786573335 | 0.483007613 | 0.412336747 | 1.171391142 | **7** | 0.655599379 | 0.858593043 | 0.562893066 | 0.39861632 | 1.412117463 |
| **9** | 0.627474282 | 0.848627771 | 0.532492102 | 0.386118023 | 1.379091548 | **9** | 0.627001472 | 0.94572053 | 0.592968164 | 0.311962 | 1.900770492 |
| **11** | 0.557773445 | 0.829167515 | 0.462487622 | 0.292606279 | 1.580579962 | **11** | 0.698894487 | 0.916614744 | 0.640616991 | 0.334270913 | 1.916460469 |
| **13** | 0.562054321 | 0.867990123 | 0.487857599 | 0.250469136 | 1.947775313 | **14** | 0.697755837 | 0.90486865 | 0.631377382 | 0.393433746 | 1.604787056 |
| **16** | 0.523405999 | 0.821870588 | 0.430171997 | 0.243171572 | 1.769006113 | **16** | 0.680990643 | 0.933813459 | 0.635918228 | 0.431012192 | 1.475406592 |
| **18** | 0.502749031 | 0.842278235 | 0.423454566 | 0.24092426 | 1.757625267 | **18** | 0.679069903 | 0.930242739 | 0.631699846 | 0.399873049 | 1.579750993 |

**Table.S2 Fractional tumor volume (FTV) after treatment with HSV-MSLN, either alone or in combination with MSLN-CAR T cells.** FTV, fractional tumor volume (mean tumor volume experimental/mean tumor volume control); E-FTV, expected FTV (mean FTV of HSV-MSLN) × (mean FTV of MSLN-CAR T cells); O-FTV, observed FTV (FTV of combination). Synergic effect: E-FTV/O-TV > 1 in red.

**Supplementary Figure 1**

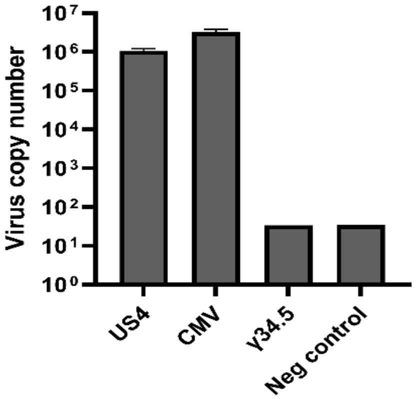


**Fig.S1 CMV-MSLN Cassette Efficiently Replaces γ34.5 in HSV-MSLN Virus.** HSV-MSLN copy number were determined by qPCR for the US4, CMV and γ34.5 genes.

**Supplementary Figure 2**

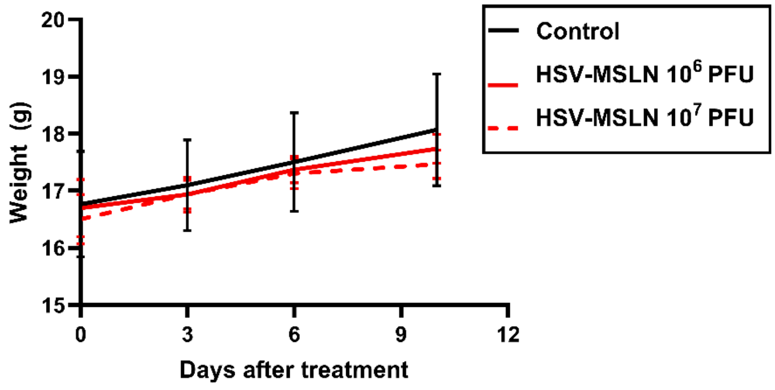


**Fig.S2 Safety of the Intratumoral Injection of HSV-MSLN in Pan02 tumor-bearing mice.** Mice weight data from the experimental design shown in fig. 2a

**Supplementary Figure 3**

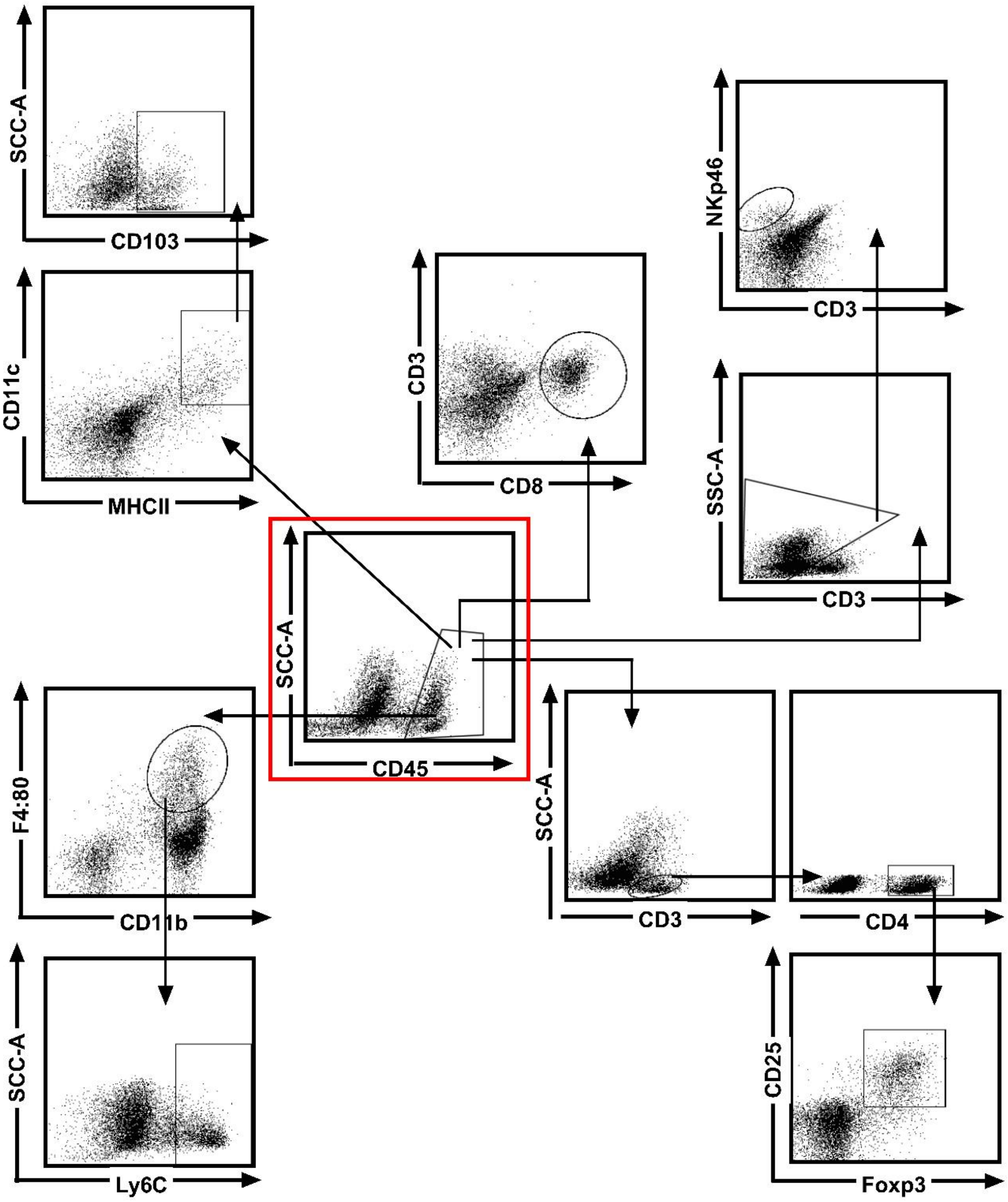


**Fig.S3 Gating Strategy of Flow Cytometry Analysis.** Gating strategy on tumor cell suspensions from Fig. 2e to check immune cell infiltration. Samples were stained with the indicated antibodies and subjected to flow cytometry analysis, and data were analyzed by FlowJo software. First, duplicates were eliminated and then gated on $CD45^{+}$ (Red square). The Shown Data were from multiple panels.

**Supplementary Figure 4**

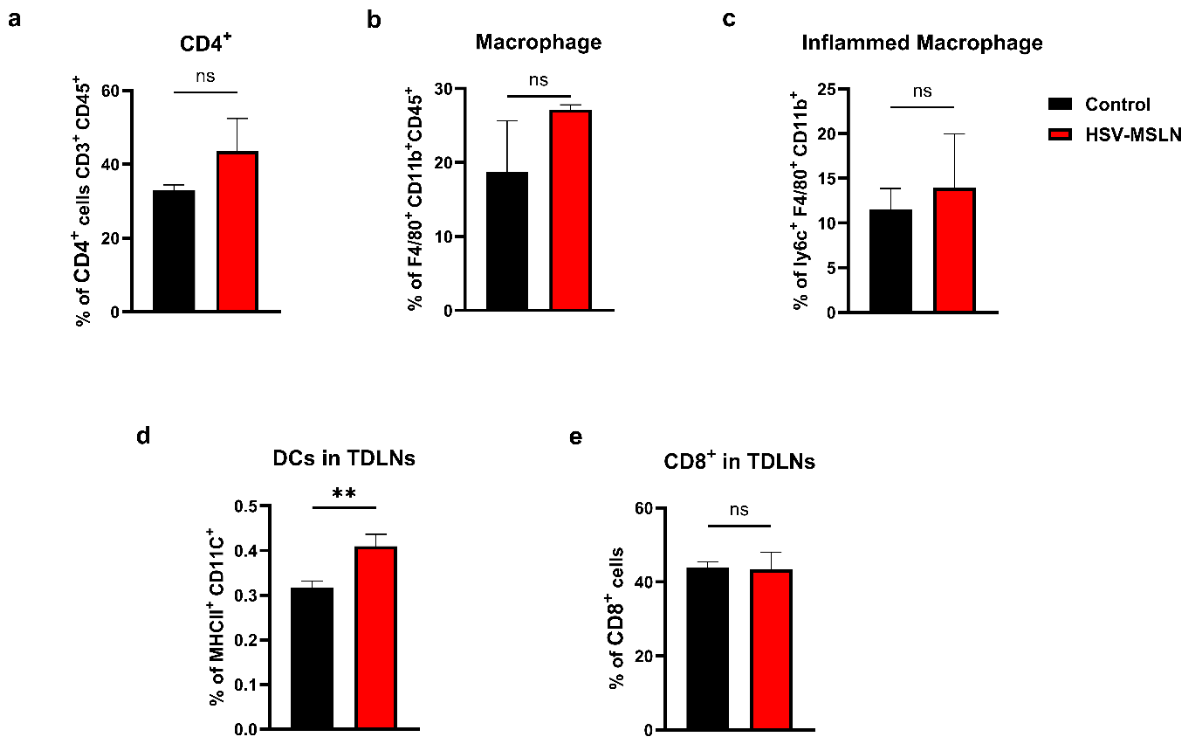


**Fig.S4 HSV-MSLN alters the immune cells infiltration is Pan02 tumor bearing mice.** The Data shown were from Fig. 2e **(a)** Bar graphs of $CD4^+$ T cells in the TME. **(b)** Bar graphs of macrophages ($F4/80^+$ $CD11b^+$ $CD45^+$) in the TME. **(c)** Bar graphs of inflamed macrophage ($Ly6c^+$ $F4/80^+$ $CD11b^+$ $CD45^+$) in the TME. **(d)** Bar graphs of DCs ($MHCII^+$ $CD11c^+$) in the TDLNs. **(e)** Bar graphs of $CD8^+$ T cells in the TDLNs. Student's t-test was performed. $^{**}p < 0.01$

**Supplementary Figure 5**

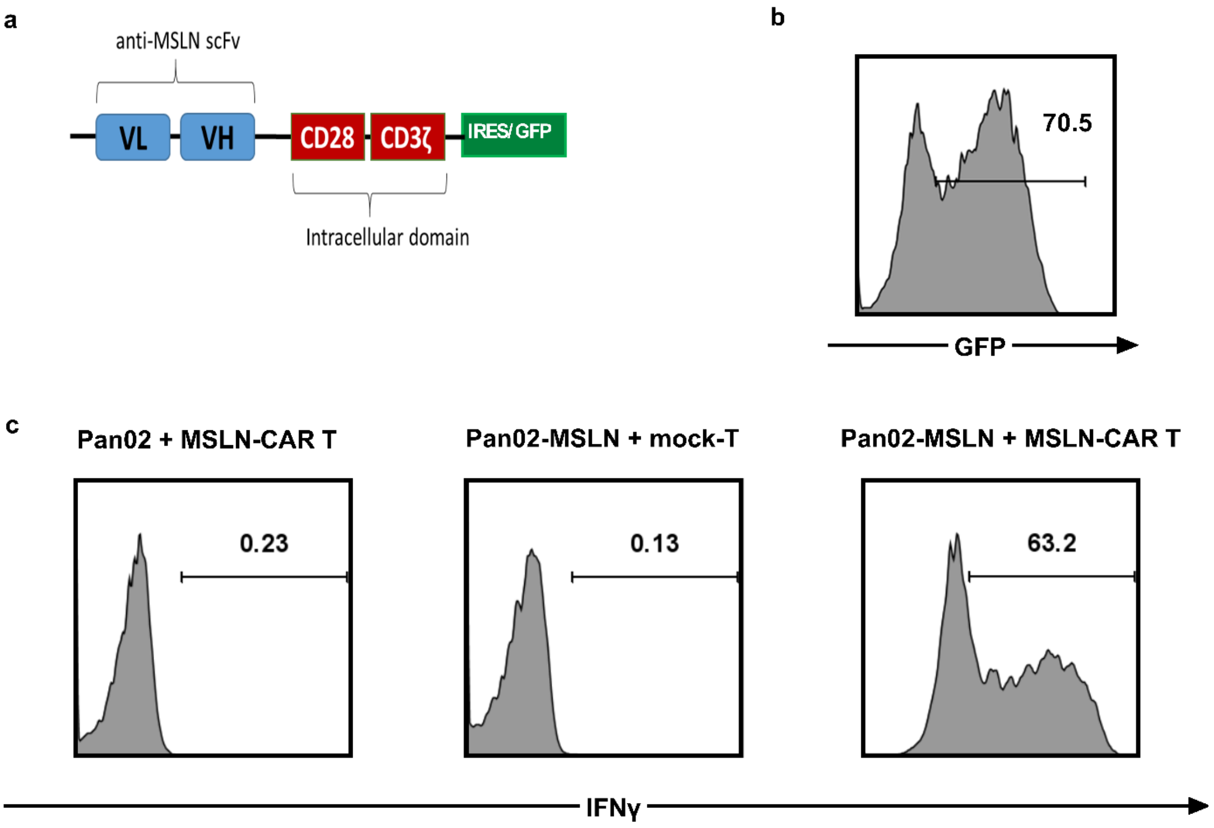


**Fig.S5 MSLN-CAR T cells exhibit activity against Pan02 cells stably expressing MSLN *in vitro*. (a)** Design of the MSLN-CAR target vector. **(b)** Representative histogram shows the percentage of $GFP^+$ CAR T cells. **(c)** Representative histograms show the percentage of intracellular $IFN\gamma^+$ from MSLN-CAR T cells cocultured with Pan02 (left), mock T cells cocultured with Pan02-MSLN (middle) and MSLN-CAR T cells cocultured with Pan02-MSLN (right) *in vitro*

**Supplementary Figure 6**

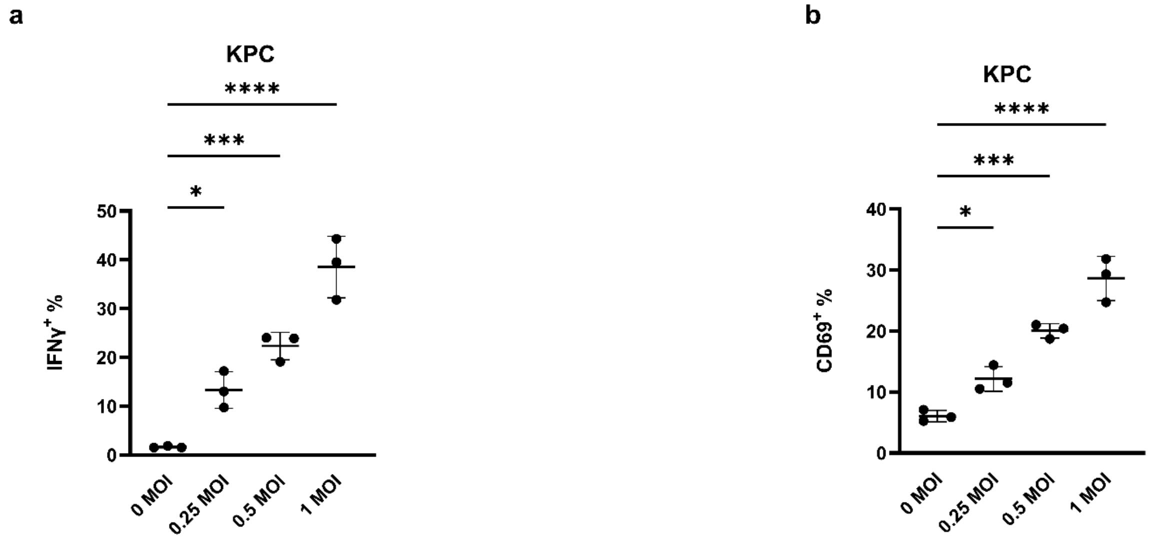


**Fig.S6 HSV-MSLN delivers MSLN to KPC cells, enhancing the activation of MSLN-CAR T cells *in vitro*.** Scatter plot graph showing the percentage of intracellular $IFN\gamma^+$ **(a)** and cell surface $CD69^+$ **(b)** in MSLN-CAR T cells after coculture with KPC infected with (0, 0.25, 0.5 and 1 MOIs) of HSV-MSLN for 6 hours. Data are presented as mean ± SD (n=3). One-way ANOVA followed by Dunnett's multiple comparison tests were performed. * $p < 0.05$, *** $p < 0.001$, **** $p < 0.0001$

**Supplementary Figure 7**

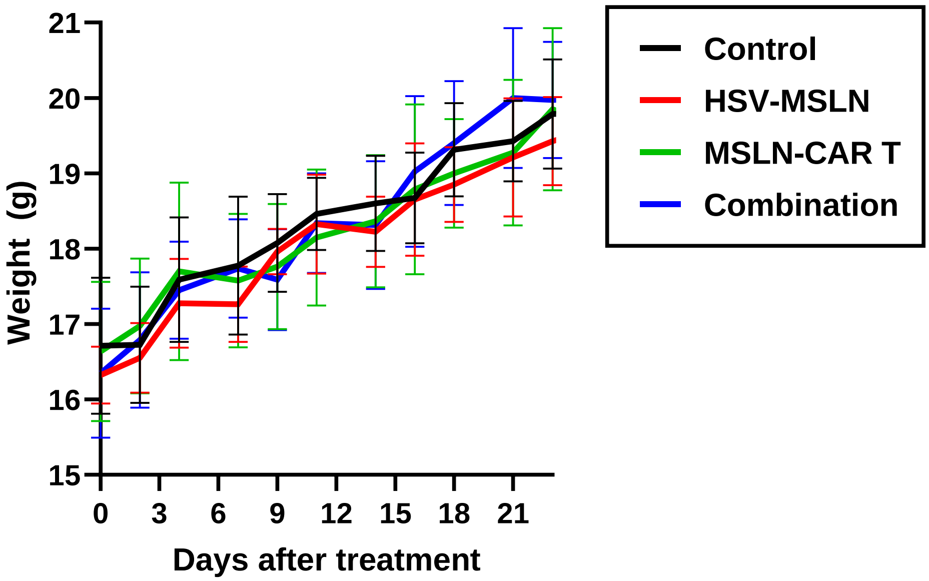


**Fig.S7 Safety of the combination therapy of HSV-MSLN and MSLN-CAR T cells in KPC tumor-bearing mice.** Mice weight data from the experiment shown in fig. 4d

## Supplementary Figure 8

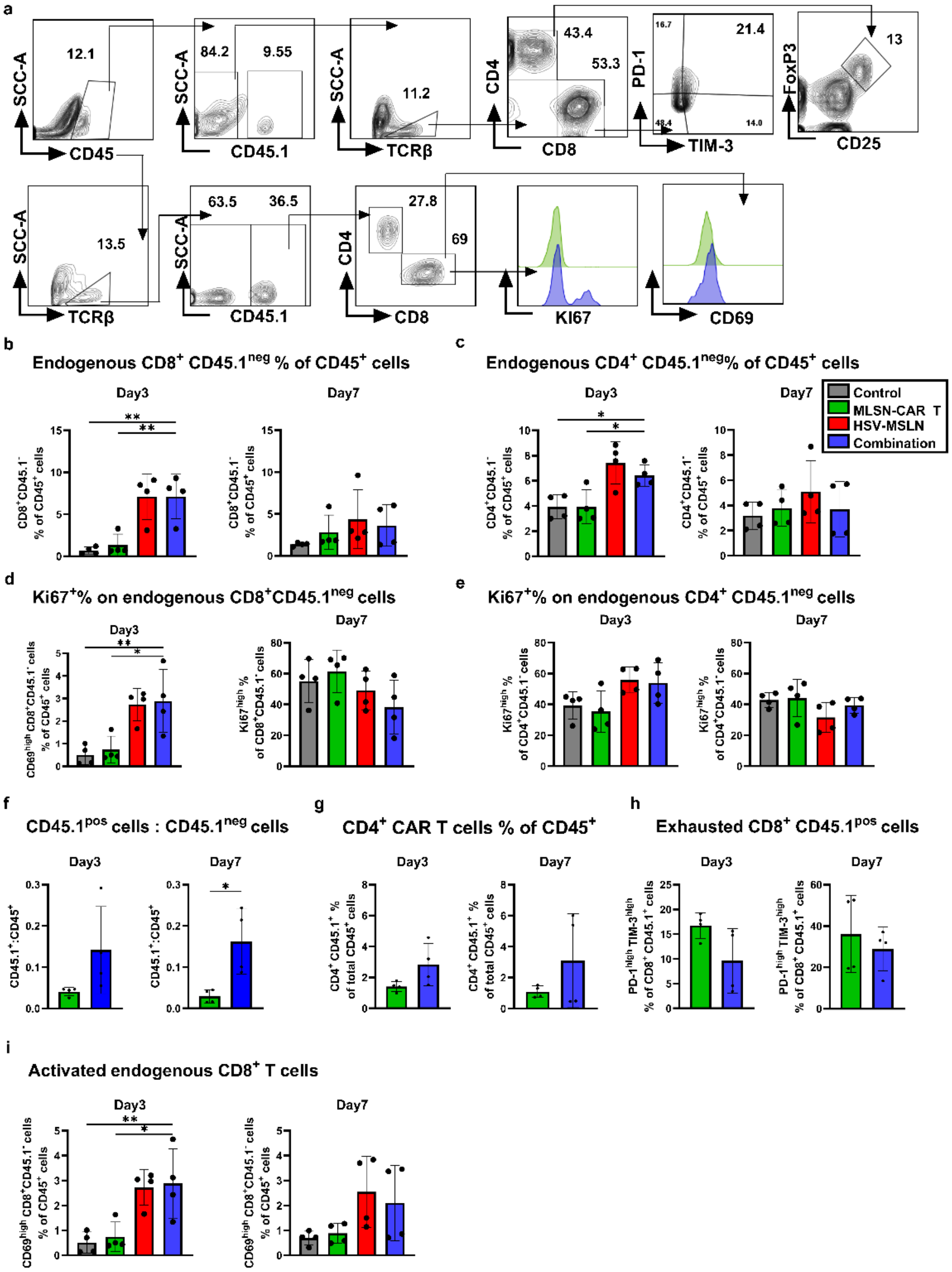


**Fig.S8 HSV-MSLN alters the immune cells infiltration is KPC tumor bearing mice. (a)** Gating strategy on tumor cell suspensions from Fig. 5 to check immune cell infiltration. **(b)** Bar graphs show endogenous CD8$^{+}$ CD45.1$^{neg}$ % of CD45$^{+}$ cells. **(c)** Bar graphs show endogenous CD4$^{+}$ CD45.1$^{neg}$ % of CD45$^{+}$ cells. **(d)** Bar graphs show Ki67 % on CD8$^{+}$ CD45.1$^{neg}$ cells. **(e)** Bar graphs show Ki67 expression on CD4$^{+}$ CD45.1$^{neg}$. **(f)** Bar graphs show CD45.1$^{pos}$: CD45.1$^{neg}$ cells ratio. **(g)** Bar graphs show transferred CD4$^{+}$ CD45.1$^{pos}$ % of CD45$^{+}$ cells. **(h)** Bar graphs show PD-1$^{high}$

TIM-3$^{high}$ expression on transferred CD8$^+$ CD45.1$^{pos}$. **(i)** Bar graphs show CD69$^{high}$ CD8$^+$ CD45.1$^-$ % of CD45$^+$ T cells. Statistical analysis was performed using one-way ANOVA followed by Dunnett's multiple comparison test. All values are presented as the mean ± SEM. *$p < 0.05$, and **$p < 0.01$

**Supplementary Figure 9**

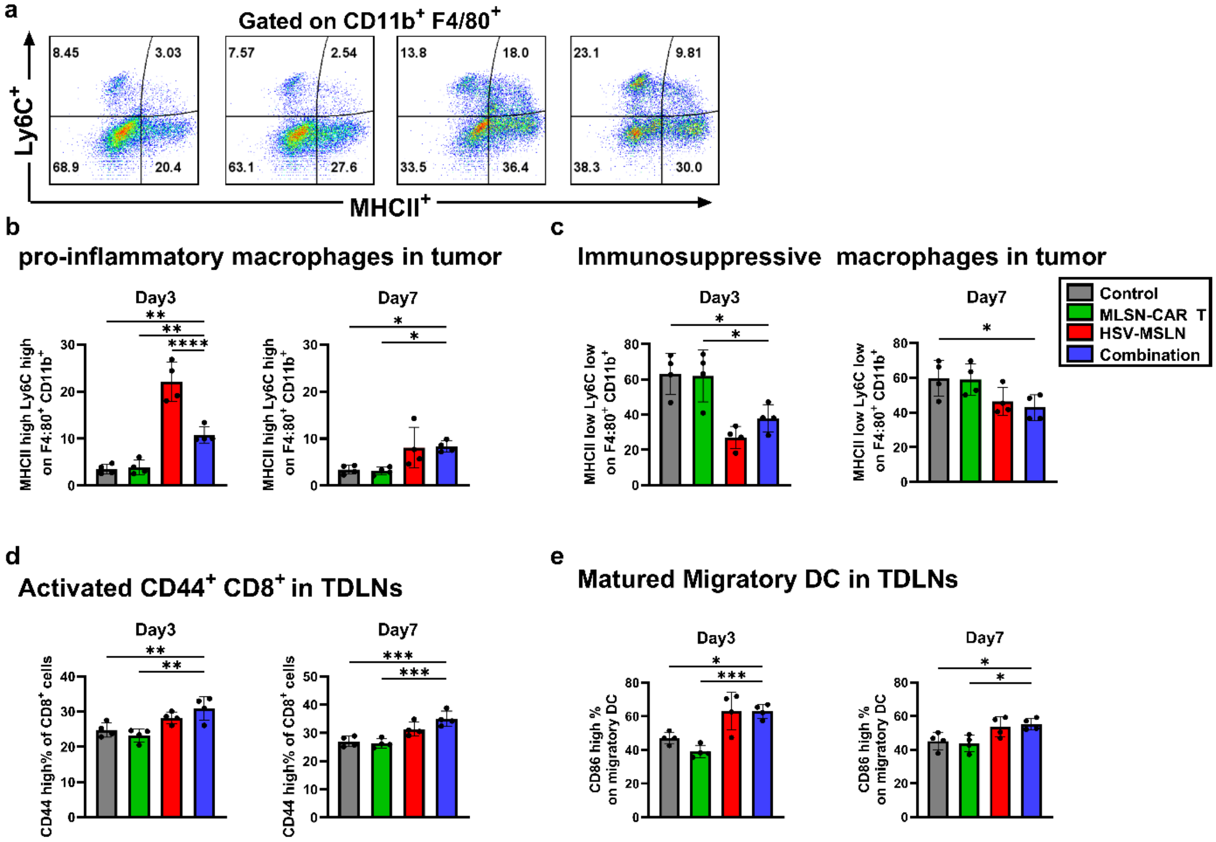


**Fig.S9 HSV-MSLN enhances Macrophage activity in TME of KPC Tumor-Bearing Mice.** Mice were subcutaneously implanted with KPC tumors and treated with two doses of HSV-MSLN ($5 \times 10^6$ PFU), followed by a single dose of MSLN-CAR T cells ($3 \times 10^6$ cells). **a-c** A single-cell suspension was prepared from tumor tissue 3 and 7 days after the last treatment and stained with indicated antibodies. (**a**) Representative dot plot of MHCII$^+$ ly6C$^+$ gated on tumor associated macrophages (CD45$^+$ MHCII$^+$ F4/80$^+$) from the four treated groups at day 3. (**b**) Bar graphs show percentages of proinflammatory macrophage (MHCII$^{high}$ Ly6c$^{high}$) in the tumor tissue. (**c**) Bar graphs show percentage of immunosuppressive macrophages (MHCII$^{low}$ Ly6c$^{low}$). (**d**) Bar graphs show percentage of CD44$^+$ CD8$^+$ T cells on TDLNs. **(e)** Bar graphs showing mature migratory DC on TDLNs. Statistical analysis was performed using one-way ANOVA followed by Dunnett's multiple comparison test. All values are presented as the mean ± SD. *$p < 0.05$, **$p < 0.01$ and ***$p < 0.001$
